# Media Multitasking: A Bibliometric Approach and Literature Review

**DOI:** 10.3389/fpsyg.2021.623643

**Published:** 2021-06-23

**Authors:** Emma Beuckels, Guoquan Ye, Liselot Hudders, Veroline Cauberghe

**Affiliations:** ^1^Center of Persuasive Communication, Ghent University, Ghent, Belgium; ^2^School of Business Administration, Northeastern University, Shenyang, China; ^3^Department of Marketing, Ghent University, Ghent, Belgium

**Keywords:** media multitasking, bibliometric analysis, content analysis, cognitive control, academic performance, advertising effectiveness, socioemotional functioning

## Abstract

Media multitasking became increasingly popular over the past decade. As this behavior is intensely taxing cognitive resources, it has raised interest and concerns among academics in a variety of fields. Consequently, in recent years, research on how, when, and why people media multitask has strongly emerged, and the consequences of the behavior for a great variety of outcomes (such as working memory, task performance, or socioemotional outcomes) have been explored. While efforts are made to summarize the findings of media multitasking research until date, these meta, and literature studies focused on specific research subdomains. Therefore, the current study adopted a quantitative method to map all studies in the broad field of media multitasking research. The bibliometric and thematic content analyses helped us identifying five major research topics and trends in the overall media multitasking domain. While media multitasking research started by studying its prevalence, appearance, and predictors, early research within the domain was also interested in the impact of this media consumption behavior on individuals' cognitive control and academic performance. Later on in 2007, scholars investigated the implications of media multitasking on the processing of media- and persuasive content, while its impact on socioemotional well-being received attention ever since 2009. Our analyses indicate that research within the field of media multitasking knows a dominant focus on adolescents, television watching, and cognitive depletion. Based on these findings, the paper concludes by discussing directions for future research.

## Introduction

Technological innovations have vitalized the high accessibility and portability of media devices, which dramatically changed the way in which people engage with media nowadays. Especially the emergence of mobile devices strongly encouraged media users to engage in media multitasking, a behavior that is defined as the simultaneous performance of multiple tasks, of which at least one is a media task (Lang and Chrzan, 2015). Many different types of behavior can be labeled as media multitasking, such as using a mobile phone while being in class, or checking emails while watching television. Figures suggest that people spend 25–50% of their media use time multitasking with media (Foehr, 2006; Voorveld and van der Goot, 2013; Segijn et al., 2017a), a number that increased year to year ever since (eMarketer, 2018). A diary study in the Netherlands to measure the prevalence of media multitasking and multiscreening behaviors suggest that more than half of the respondents reported a simultaneous use of multiple screens at least once in the measured week (Segijn et al., 2017a). These respondents indicated to spend on average 30 min a day multiscreening. Although people across all ages are fervent media multitaskers, younger people tend to multitask more often (Brasel and Gips, 2011; Segijn et al., 2017a). For adults, 24% of the media day involves consuming multiple media simultaneously, while for young people, 29% of the media day involves using more than one medium concurrently (Voorveld and van der Goot, 2013). Besides, it is argued that children engage in media multitasking starting from a very young age, as one third of 3–4 year olds are already multitasking with media devices (Kabali et al., 2015).

Along with the growing tendency to multitask with media, academic interest in the phenomenon increased steadily over the last years. Ever since the first study on media multitasking behavior was published in 1990 (Armstrong and Greenberg, 1990), academic research into the topic was flourishing across domains and disciplines. The current study therefore aims to provide an overview of the research on media multitasking across different research fields to discover trends and identify research gaps. This will provide guidance for setting out a future research agenda. Much of the research in the domain of media multitasking focuses on performance and found that people are actually incapable of parallel processing, whereby they sequentially and quickly shift and distribute their attention between several tasks instead (Srivastava, 2013; Miller, 2017). Various neuropsychological studies have been showing that this multitasking behavior is highly mentally taxing, with detrimental effects on cognitive outcomes such as working memory capacity, long-term memory, task switching, and filtering of irrelevant information (Strobach et al., 2014; Medeiros-Ward et al., 2015; Uncapher et al., 2016).

However, there are some studies that find no such an effect (Ophir et al., 2009; Baumgartner et al., 2014; Ralph et al., 2014), and even find positive effects on task switching (Alzahabi and Becker, 2013), attention control (Cardoso-Leite et al., 2016), and ability to split attention (Yap and Lim, 2013). These mixed findings may result from the differences in media multitasking contexts that previous studies focused on. The juggling with different media tasks has been shown to be particularly cognitively demanding, especially when people have low user control over the media (Jeong and Hwang, 2016), the two media tasks share sensory channels (Jeong and Hwang, 2015) or involve multiple sensory channels (Wang et al., 2015), the media tasks are unrelated (Wang et al., 2015) and have a distant physical proximity (Jeong and Hwang, 2016). Given the occurrence of media multitasking in many different situations, people's cognitive resources are highly being taxed many times a day. Furthermore, as the impairment of cognitive resources has often been considered as a risk factor for various memory, behavioral, and impulse-control outcomes (Heatherton and Baumeister, 1991; Lyon and Krasnegor, 1996; Baumeister et al., 2007), media multitasking behavior has become the subject of academic research in a wide range of disciplines over the past years.

The intense and rapid growth of studies approaching this specific type of media behavior from different research perspectives, makes the overall view on the research domain hard to grasp. While initial and valuable efforts have been made to summarize the findings of previous media multitasking research, these meta-analyses or literature reviews mostly focused on one subdomain of media multitasking research (e.g., advertising effectiveness, Segijn and Eisend, 2019; or cognitive control, Wiradhany and Nieuwenstein, 2017). However, the increasing interest from various fields, like cognitive psychology, developmental psychology, educational sciences, marketing, communication sciences, etc. in media multitasking behavior and its consequences for people's mental processes, performance, and functioning calls for an approach to map this research field from a multidisciplinary perspective. Combining the research results and insights across these different disciplines may benefit the theoretical development of media multitasking as a phenomenon that distinguishes itself from more general multitasking.

Hence, the current study fills this gap by adopting a bibliometric approach, aiming to capture a variety of article information and to connect this information in a quantitative way to assess the evolution, main journals and authors, and the impact and diffusion of the research studies within a broader research field. Additionally, based on (co-) keyword analysis and the (qualitative) in-depth review of the content of the included studies (281 in total), five different research topics came to the foreground, in which studies by researchers from various academic subdomains are combined. Furthermore, the evolution of studies is described within each research topic as well. Moreover, a substantial merit of the analyses of the subject's matter, is that it allows to detect the collaborations and/or thematic overlap among the different subdomains. The results of these analyses led to the identification of research gaps and potential future research opportunities. We believe that this approach offers an exhaustive, multidisciplinary and objective overview on the research that is specifically interested in today's ever growing digitalization and people's consequent chronic media consumption behavior.

## State-of-the-Art of Media Multitasking Research

Although research on media multitasking has only known a steep increase in academic attention in recent years, many researchers in various disciplines pooled the insights of the multitude of studies in literature reviews and meta-analyses. Additionally, conceptual papers providing premises and underlying explanations to investigate in further research, accelerate the progression in this research area. Without questioning the added value of each of these overview articles, a bibliometric study across the different research fields and disciplines within the broad field of media multitasking, will contribute to the current state-of-the-art by providing a wide view on the field and this for various dependent variables such as learning and performance or socioemotional functioning. One recently published study specifically adopted a bibliometric analysis method to provide an insight into the research domain of multitasking in various contexts, but not specially related to multitasking with media use (Rózańska and Gruszka, 2020). This review sheds a light on the research trends found in 324 multitasking studies published between 2000 and 2018 based on a keyword analysis. This work provides relevant insights, but is not precise in giving an overview of studies related to “media” multitasking, as a specific and distinctive form of multitasking.

Based on our literature search, 17 papers provide an overview of literature and research on media multitasking. Most of these papers are conceptual in nature and focus on the psychological mechanisms explaining performance deficits in a multitasking context (see Carrier et al., 2015; Uncapher et al., 2016; Lin and Parsons, 2018; Aagaard, 2019; Duff and Segijn, 2019). Lin and Parsons (2018), for instance, provide insights into the conflicting findings in past research regarding the impact of media multitasking on cognitive control and learning outcomes. They integrate theoretical and empirical findings of different disciplines to provide a more in-depth understanding of the consequences of the phenomenon.

Further, several systematic literature reviews were conducted to provide an insight into the impact of media multitasking on task performance and the role of low levels of cognitive control and executive functioning deficits (Lang and Chrzan, 2015; van Der Schuur et al., 2015; May and Elder, 2018; Parry and le Roux, 2018). Compared to the conceptual literature reviews, these papers are demarcated by their focus on specific target groups, theories, or domains. For example, van Der Schuur et al. (2015) specifically focus on the consequences of media multitasking behaviors for adolescents (12–18 years), while May and Elder (2018) reviewed studies on the impact media multitasking behavior has on academic performance. Parry and le Roux (2018) focus in their review on the success of different types of interventions (e.g., mindfulness) that may decrease the detrimental impact of media multitasking on attention-related task performance.

Next to these conceptual and review studies, five meta-analyses are published that provide insights on the robustness of the effects of media multitasking on performance (Kämpfe et al., 2011; Jeong and Hwang, 2016; Wiradhany and Nieuwenstein, 2017; Segijn and Eisend, 2019; Wiradhany and Koerts, 2019). As such, Kämpfe et al. (2011) performed a meta-analysis on 189 studies examining the impact of background music and suggests that the overall null effect that was found can be explained by contrasting effects on differential outcomes (e.g., detrimental effect on reading while positively affecting sports performance). Wiradhany and Koerts (2019) and Wiradhany and Nieuwenstein (2017) conducted meta-analyses (*N* = 15 and *N* = 39 studies, respectively) to examine the impact of media multitasking on attention regulation. The meta-analysis of Jeong and Hwang (2016), including 49 media multitasking studies published before 2014, further identified different moderating factors, such as task relevance impacting the effects of media multitasking on cognitive and attitudinal outcomes. To conclude, in their meta-analysis of 29 datasets, Segijn and Eisend (2019) specifically focused on advertising effects in a media multitasking context.

The current study contributes to the insights gained in media multitasking studies by focusing on all articles related to the full domain of media multitasking, by transcending the boarders of specific academic subdomains. We believe this will provide interesting insights as it will enable us to map the research both within and between different sub fields of media multitasking. Thus, the purpose of the current study is to provide a systematic review in the field of media multitasking. More specifically, this paper will examine (1) which journals and scholars are active in, contribute more to and have the most impact within this field; (2) which focus do media multitasking studies have, how are the topics related to each other and how did they evolve over time; and (3) what are the current research gaps in media multitasking research and which research paths are interesting for future studies. To solve these questions, both bibliometric and thematic content analyses were adopted and the results will enable future scholars to see where the field began and trace its shift over time (Andriamamonjy et al., 2019; Caff et al., 2020). Before reporting the results of our mapping approach, we explain the specific methodology used in this review.

## Methodology

We start this section by explaining the procedure of paper selection and refinement. Afterwards, we explain how we analyzed the content of the different articles.

### Paper Selection Procedure

To identify relevant papers for the bibliometric analysis, we used the same procedure as used in similar bibliometric studies (Bartolini et al., 2019; Guo et al., 2019). First, the Scopus database was selected to search for relevant literature. The reasons behind this are 2-fold. First, Scopus is the largest multi-disciplinary database of science, technology, medicine, social science, and arts and humanities, which is useful for mapping a smaller and multi-disciplinary research field as media multitasking research (Feng et al., 2017; Kolle et al., 2018). Second, the database provides various document data formats allowing bibliometric software to process it conveniently.

Second, the keywords were defined to detect the appropriate studies in Scopus, using the “title-abstract-keyword” search. Based on the search strategies used in previous media multitasking literature reviews in various research domains (van Der Schuur et al., 2015; Jeong and Hwang, 2016; Segijn and Eisend, 2019), relevant keywords were identified. In particular, we adopted two subsequent search strategies. First, we searched for relevant papers by combining the keywords “media” and “multitask^*^” using the “AND” Boolean logic. Second, in a new search activity, some separate strings of keywords were added including “multiscreen^*^,” “multi screen^*^,” “dual screen^*^,” “cross screen^*^” and “second screen^*^” using the “OR” logic. The specific search formulas were as follows: formula 1: “media” AND “multitask^*^”; formula 2: “multiscreen^*^” OR “multi screen^*^” OR “dual screen^*^” OR “cross screen^*^” OR “second screen^*^.”

**Figure 1:**
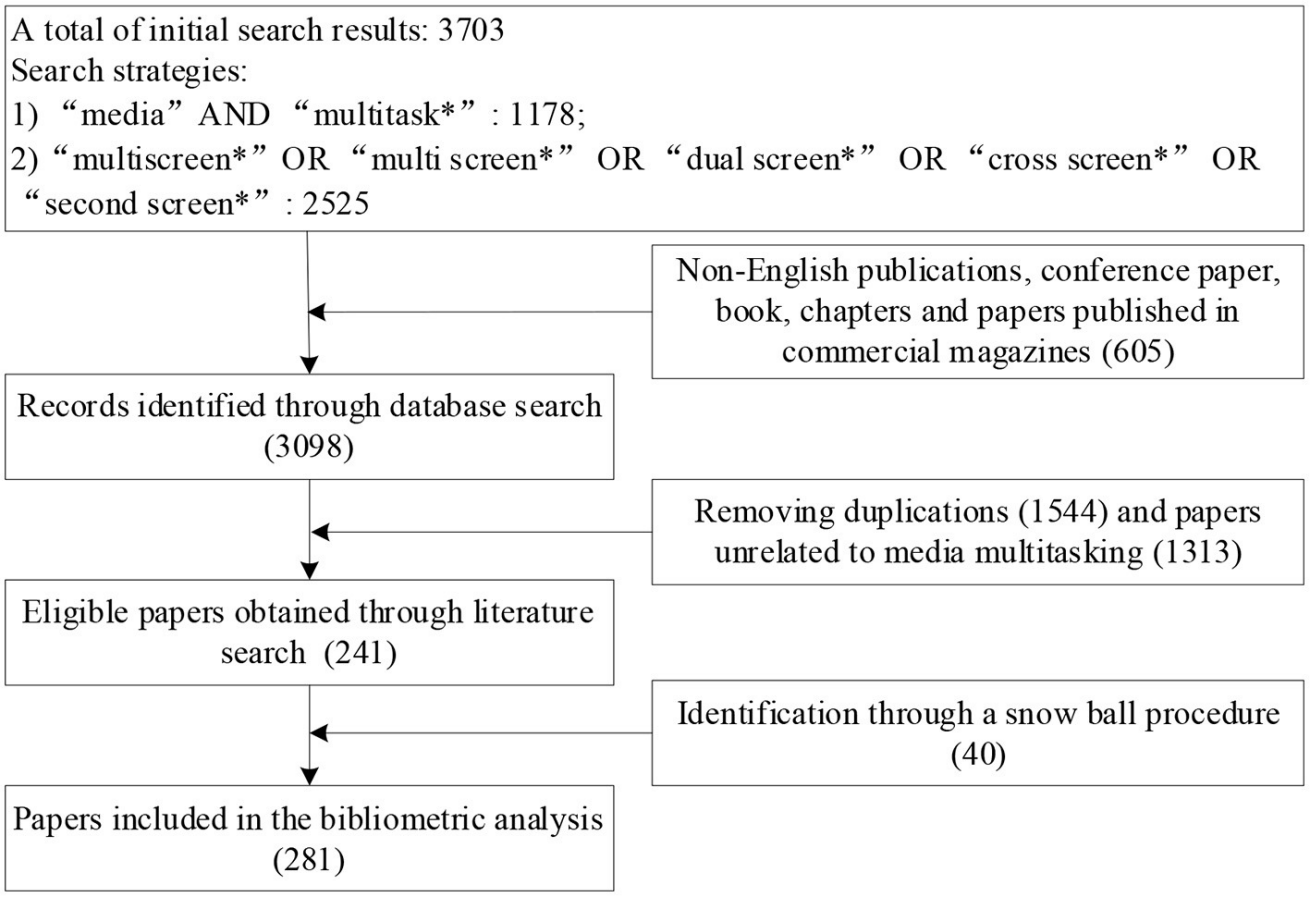
Literature search and refining criteria for bibliometric analysis.

These keywords were then used to find all media multitasking research published before September 2020. A total of 3,703 articles were collected in this initial search and the results were saved in a RIS format. That way, each article included all the necessary information for subsequent analysis, such as title, abstract, author(s), keywords, and references. This initial search included various document types that were written in a variety of languages. To guarantee the quality of the papers included in data analysis, we focused only on full-length and peer-reviewed articles and therefore did not include conference proceedings and books (Shen and Ho, 2020). Considering English is the most common language in research, we decided to include only papers written in English (Guo et al., 2019). Moreover, as the initial search was conducted by using two search formulas, there were many duplicates that needed to be removed. After this screening process, it had to be noted that many of the collected papers were not related to the area of media multitasking as the search formula used in this study is “‘media’ AND ‘multitask^*^’” but not “media multitask^*^.” Accordingly, this led to the inclusion of many papers which were actually not related to media multitasking topics (e.g., Habic et al., 2020). Thus, the first two authors of this manuscript reviewed the remaining papers' titles, abstracts and main texts to determine whether they were related to the research topic of media multitasking and papers were included only if they focus on media multitasking topics. As for doubtful cases of inclusion, there was a discussion between the authors to decide upon the inclusion or exclusion of the article. This procedure left us with a total of 241 articles. To ensure that our search process did not miss any relevant articles, the reference lists of these studies were further inspected to find additional articles which were not yet included in the sample. This led us to the identification of an additional 40 articles which were relevant for this review. Accordingly, a total of 281 papers were collected to map the evolution of media multitasking research. A more detailed overview of the literature search and refining process can be seen in Figure 1.

### Data Analysis Procedure

First, in the *bibliometric analysis*, the evolution of published studies throughout the years is mapped and the most prolific journals and authors are examined based on a descriptive analysis. The software tool BibExcel was used to extract the relevant information (title, abstract, keywords, and references). This tool is compatible with the visualization tool VOSviewer that we used to visually present the results of our analysis.

Second, a *thematic content analysis* was conducted to identify research clusters and trends in the research on media multitasking based on keyword and co-word analyses and an in-depth investigation of the content of the studies. The most frequently used words or phrases in the papers' titles and keywords were obtained using BibExcel and the co-occurrence of these keywords was visualized using VOSviewer. These analyses enabled us to identify the research topics in past media multitasking research. Each paper was carefully read and coded. These codes were then further used to categorize the papers in relation to the research topics. A joint content analysis was then conducted to examine the interrelations between the different subdomains and a dynamic content analysis has been performed to map the evolution of the different subdomains throughout the years.

## Descriptive Results of Bibliometric Analysis

### Trends in Media Multitasking Research

**Figure 2:**
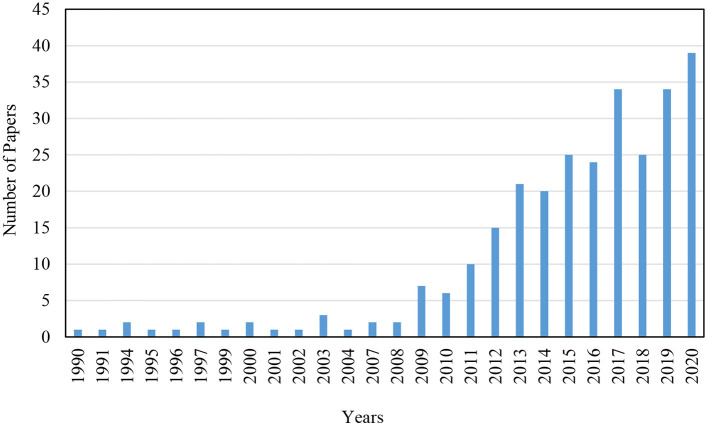
Number of publications on media multitasking over the years.

The first media multitasking article was published in 1990, which is relatively early, knowing that the topic did not receive much further research attention until 2011 (see Figure 2). Accordingly, the development of media multitasking research can be divided into an initial and a growth stage. In the initial stage (from 1990 to 2010), <10 media multitasking articles were published annually. In the growth stage (from 2011 to 2019), an explosive growth in number of media multitasking publications can be witnessed. This period represents about 90.04% of the analyzed papers within this study. Although the number of media multitasking publications has known a small decrease in 2018, the general trend of media multitasking research was one of rapid growth. Although the search was limited to papers published until September 2020, this review reveals that there is again an increase in media multitasking research published in 2020 compared to 2019.

The selected 281 media multitasking articles appeared in 129 different journals. Table 1 provides an overview of the ten most contributing journals to the field of media multitasking research. A total of 118 articles were published in these ten journals, representing 41.99% of all articles. In particular, the journal with the most publications related to the topic of media multitasking is *Computers in Human Behavior* with 53 articles (about one fifth of the articles in the sample), followed by *Computers and Education, Journal of Advertising*, and *Media Psychology*. When looking at the disciplines of the journals which are publishing media multitasking studies, communication (11%), general psychology (9.4%), and educational sciences (8.6%) are most prolific. Journals covering other disciplines, such as cognitive neuroscience (0.5%) or health (0.1%), contribute less often to media multitasking research.

**Table 1:** The ten most productive journals contributing to media multitasking research.

**Journal name**	**Subject**	**Total number**
Computers in Human Behavior	General psychology	53
Computers and Education	Education	18
Journal of Advertising	Communication	9
Media Psychology	Applied psychology	8
Attention Perception and Psychophysics	Experimental and Cognitive Psychology	6
Human Communication Research	Communication	6
Psychonomic Bulletin and Review	Experimental and Cognitive Psychology	5
Journal of Broadcasting and Electronic Media	Communication	5
Journal of Communication	Communication	4
Cyberpsychology, Behavior, and Social Networking	Communication	4
Total		118

A total of 547 different authors could be identified in the media multitasking studies we analyzed. The large majority of authors (80%) published only one media multitasking study, whereas the remaining 20% (114 authors) published at least two papers that were included in our sample. As Table 2 shows, Segijn published the highest number of media multitasking studies, followed Jeong, Kononova, Voorveld and Baumgartner looking at the ratio of media multitasking publications vs. the total number of publications an author has, analyses suggest that Segijn, Kononova and Ralph devoted most of their research attention to the field of media multitasking. These researchers are active in the broad field of (persuasive) communication. Segijn, Voorveld and Smit specifically focus on the advertising domain, while Smilek and Ralph examine the consequences of media multitasking on attention and cognition. Other prolific authors, including Jeong, Kononova, Baumgartner and Lin focused on a variety of topics, such as learning and academic performance, predictors of media multitasking behavior, and differences in media multitasking behavior across countries and generations.

**Table 2:** The ten most prolific authors contributing to media multitasking research.

**Authors**	**Current affiliation**	**Number of publication**	**Total publication**	**Ratio**
Segijn C.M.	University of Minnesota, USA	11	17	64.71%
Jeong S.H.	Korea University, Korea	9	35	25.71%
Voorveld H.A.M.	University of Amsterdam, Netherlands	9	34	26.47%
Kononova A.	Michigan State University, USA	8	21	38.10%
Baumgartner S.E.	University of Amsterdam, Netherlands	8	28	28.57%
Hwang Y.	Myongji University, Korea	7	31	22.58%
Smilek D.	University of Waterloo, Canada	7	149	4.71%
Ralph B.C.W.	University of Waterloo, Canada	7	20	35.00%
Lin L.	University of North Texas, USA	6	49	12.24%
Smit E.G.	University of Amsterdam, Netherlands	6	83	7.23%

*This table only considers papers published until September 2020*.

Authors currently affiliated to the University of Minnesota, Korea University, University of Amsterdam, Michigan State University, Myongji University, University of Waterloo and University of North Texas contributed the most to media multitasking research.

### Identification of Impactful Authors and Publications Within the Media Multitasking Domain

A citation analysis (local and global citation times) was used to identify the most influential authors (cf. Table 3) and publications (cf. Table 4) in our sample. The local citation time refers to the number of citations within the study's sample, while the global citation time was assessed by checking the number of citations in the Scopus database. Hence, the discrepancy between the global and local citation index refers to the impact a paper or author has in other domains than media multitasking research. Additionally, authors' local *h*-index was explored which refers to an author's number of media multitasking papers (*h*) that have each been cited at least (*h*) times by other media multitasking studies. This index gives an insight into the quantity (in terms of number of studies in the domain) and quality (in terms of impact on other scholars) of an author's media multitasking publications. To measure the impact (in terms of shares, discussions, and likes) of the media multitasking research on society, the altmetric score was used (obtained from https://www.altmetric.com/). This score gives insight into the number of mentions in online media such as Facebook, Mendeley, Twitter, and Wikipedia.

**Table 3:** The ten most cited authors in media multitasking area.

**Authors**	**Local citation times**	**Global citation times**	**Local *h*-index**	**Altmetric score**
Jeong S.H.	208	383	7	8
Nass C.	169	871	2	1,109
Rosen L.D.	167	804	5	296
Wagner A.D.	162	789	3	1,591
Cheever N.A.	158	777	4	258
Carrier L.M.	158	777	4	258
Ophir E.	134	698	1	1,069
Wang Z.	127	301	4	249
Hwang Y.	112	204	5	8
Fishbein M.	110	198	3	0

*This table only considers papers published until September 2020*.

**Table 4:** The ten most cited media multitasking papers.

**Publications**	**Local citation times**	**Global citation times**	**Altmetric score**	**Research topic**
Ophir et al. (2009)	134	698	1,069	Differences in information processing styles between heavy and light media multitaskers
Carrier et al. (2009)	67	184	23	Differences in media multitasking across three generations of Americans
Jeong and Fishbein (2007)	66	135	0	Predictors of media multitasking behavior
Brasel and Gips (2011)	62	148	18	The switching behavior during concurrent television and computer usage
Wang and Tchernev (2012)	55	172	91	The cognitive and emotional effects of media multitasking
Bowman et al. (2010)	52	193	22	Effects of media multitasking (instant messaging) on reading performance
Hembrooke and Gay (2003)	47	270	163	Effects of media multitasking (laptop) in lecture
Rosen et al. (2013)	47	291	194	Factors of media multitasking during studying
Alzahabi and Becker (2013)	44	79	15	Comparison of heavy and light media multitaskers in attention, working memory, task switching, and fluid intelligence, as well as self-reported impulsivity and self-control
Minear et al. (2013)	43	75	14	Comparison of heavy and light media multitaskers in task-switching and dual-task performance

*This table only considers papers published until September 2020*.

The results of this analysis reveals that Jeong obtained the highest number of citations in our sample and can be considered the most influential scholar in the media multitasking domain. Of all prolific authors identified in Table 2, he also has the highest local h-index, followed by Hwang. Interestingly, some of the other prolific authors that published more than two paper on the topic Segijn, Voorveld, Kononova and Smit were not yet highly cited authors. A plausible explanation for this is that some of their papers were published in more recent years and had less time to accumulate citations. Furthermore, the high local and global citation indices of Nass, Rosen, Wagner, Cheever, Carrier and Ophir indicate that their publications were not only frequently cited within other media multitasking articles, but also by papers in other disciplines. In addition, the high altmetric score of Nass, Wagner and Ophir suggest that their publications were often discussed and shared online.

The most influential publication in our sample, both in terms of local and global citation index and in altmetric score was that of Ophir et al. (2009). This paper presents a measure for people's media multitasking habitual behavior distinguishing heavy from light media multitaskers, the media multitasking index. This pioneering study, showing that heavy media multitaskers performed worse on a set of cognitive control performance tasks compared to light media multitaskers, was often referred to and replicated by subsequent research, both in the field of media multitasking, but also in other domains. In addition, the studies of Carrier et al. (2009), Jeong and Fishbein (2007), Brasel and Gips (2011), Wang and Tchernev (2012), Bowman et al. (2010) were also regarded as highly influential papers due to their great amount of local and global citation times. These papers cover a wide range of topics ranging from examining how different generations coped with at-home multitasking situations (Carrier et al., 2009) to examining what motivates people to perform media multitasking behaviors (Wang and Tchernev, 2012) and which media and audience factors predict this media multitasking behavior (Jeong and Fishbein, 2007).

## Identifying Trends in Media Multitasking Research

### Identifying Research Topics With a Keyword and Co-Word Analysis

**Figure 3:**
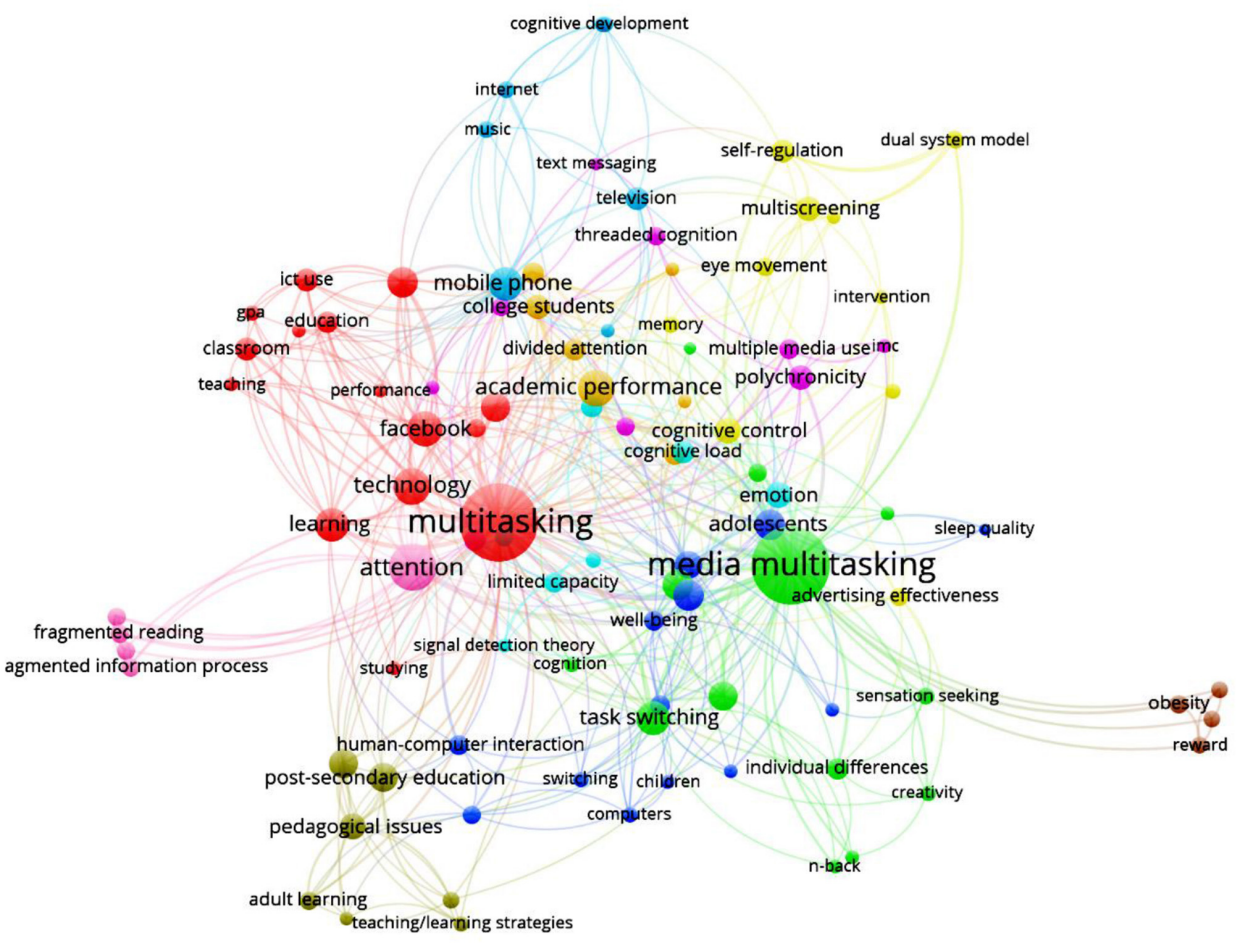
The visualization of co-word analysis.

An analysis of the title and keywords fields revealed a total of 909 title words which occurred 2,410 times and a total of 605 keywords which occurred 1,138 times in our sample. All title and keywords were then manually screened to group words with similar or identical meaning (e.g., “media multitasking” and “media-multitasking”). Table 5 shows the 20 words which occurred most often in title or keyword fields. A visualization of the keywords that often appear together (i.e., co-word analysis) can be found in Figure 3. This analysis was performed using VOSviewer and explores which keywords often appear together. Each node represents an independent keyword, and the size of the nodes is proportional to the frequency in which this keyword appeared in the studies. The lines between the nodes indicate that the two connected keywords appear together in papers, and the thickness of these lines represents the frequency of their co-occurrence.

**Table 5:** The 20 most frequently used words in paper titles and keyword field.

**Words in paper titles**	**Frequency**	**Words/phrases in keywords**	**Frequency**
Multitasking	196	Media multitasking	89
Media	167	Multitasking	62
Effects	68	Attention	21
Screen	39	Academic performance	14
Performance	38	Adolescents	14
Learning	35	Second screen	14
Television	33	Distraction	12
Students	32	Texting	12
Attention	26	Learning	11
Task	26	Television	10
Classroom	25	Technology	10
Distraction	23	Mobile phone	10
Adolescents	22	Post-secondary education	9
Study	21	Media in education	9
Cognitive	17	Working memory	9
Academic	17	Task switching	8
Reading	16	Facebook	7
Relationship	16	Cognitive control	7
Advertising	14	College students	7
Background	13	Advertising effectiveness	6

From Figure 3, it can be inferred that node sizes of “media multitasking,” “multitasking,” “attention,” “learning,” “television,” “task switching,” “technology,” “academic performance,” “adolescents,” and “mobile phone” are bigger than the other keywords, indicating high focus on these topics. The analysis further shows that all keywords can be grouped into eleven clusters (cf. 11 different node colors in Figure 3), which can be further grouped into five thematic research topics through a cluster labeling process. The first research topic incorporates studies in cluster 1 reflecting keywords as “uses and gratifications,” “media and technology,” “mobile phone,” and “Internet.” Thus, it can be inferred that it involves research on people's motives to perform media multitasking behaviors and the identification of variables that may predict the occurrence of this behavior. The second research topic groups clusters 2, 3, and 4 as they all focus on keywords related to attention and cognition: “working memory,” “executive functions,” “cognitive flexibility,” “metacognition,” “threaded cognition,” “cognitive load,” “limited capacity,” and “recognition memory.” This research focuses on the impact of media multitasking on cognitive outcomes. The third topic covers clusters 5–8, which all reflect studies on learning and academic performance (with keywords as “media in education,” “learning,” “studying,” “academic performance,” etc.). The fourth topic reflects the studies in cluster 9 on advertising effects and the processing of media content in global (“advertising,” “memory,” “evaluation,” “visual attention”, etc.). The fifth topic combines studies in cluster 10 and 11, in which the keywords “children,” “adolescents,” “obesity,” “self-control failure,” “well-being,” and “sleep quality” were common. These clusters suggest that researchers have showed interest in the effects of media multitasking on people's socioemotional functions. Below, we will discuss the most important insights of the research published in each of the research topics. Given the fact that some articles are related to topics across different research themes, we discuss them into multiple topics (see Appendix A for an overview of the research topics and the studies in those topics).

### Topic One: Motivating and Predicting Media Multitasking Behaviors

Until date, many researchers attempted to detect the antecedents and motivations of engaging in media multitasking behavior. Various characteristics related to the media and audience, such as socio-demographic factors (Srivastava et al., 2016), media ownership (Jeong and Fishbein, 2007), personality traits (Jeong and Fishbein, 2007), and media multitasking gratifications (Wang and Tchernev, 2012), have been investigated to better understand why people increasingly multitask with different media today. For example, the study of Voorveld and van der Goot (2013) revealed that media multitasking habits were common across all age groups, but that different generations distinguish themselves from each other in terms of preferred media combinations. Particularly, younger age groups often combine music with online activities, whereas the older age groups are more inclined to listen to the radio while simultaneously engaging with their e-mails or reading a newspaper. Besides, other research also shows that younger generations tend to experience less difficulties when multitasking compared to older generations (Carrier et al., 2009). In addition, it is argued that individuals' ethnicity and country-of-origin also affects their media multitasking preference, which can be explained by economic, political and cultural characteristics varying across countries (Kononova et al., 2014; Kononova and Chiang, 2015). For example, Russian students showed a significant smaller tendency to media multitask compared to students from the U.S. and Kuwait. A possible explanation for this finding could be their smaller media device ownership due to lower income per capita, poorer information communication technology market and political developments in Russia compared to the U.S. and Kuwait (Kononova et al., 2014). Hence, it is argued that individual differences affect people's media multitasking tendency and behaviors.

### Topic Two: Media Multitasking and Cognitive Outcomes

Engaging in media multitasking is cognitively demanding as it requires people to switch tasks, prioritize, and schedule those tasks (Sanjram, 2013). These actions require individuals to focus their attention, neglect irrelevant information, and allocate attentional resources to the different tasks. Accordingly, media multitasking involves a heavy cognitive workload as the processing of multiple streams of information is highly demanding (Sanjram, 2013). However, it is argued that human beings only have a limited amount of resources available at a certain moment in time, whereby engaging in two tasks instead of one depletes those limited pools of resources more quickly (Lang, 2000). Following up on the pioneering study of Ophir et al. (2009), which distinguished heavy from light media multitaskers based on people's tendency to multitask with media, many studies showed interest in the relation between media multitasking frequency and cognitive control outcomes. For example, Uncapher et al. (2016) found that chronic media multitaskers are associated with higher attentional impulsivity. Besides, the study of Cain et al. (2016) also suggested that frequent media multitasking behavior is not only associated with poorer executive functioning, but also with a reduced growth of mindset. The latter refers to people's belief whether their intelligence or ability was malleable and could grow or be improved with effort, rather than being a set factor beyond their control and is associated with better academic achievement (Dweck, 2006; Blackwell et al., 2007). In the same line, Baumgartner et al. (2014) found that adolescents who media multitask more frequently have more problems with performing executive function control in terms of working memory capacity, the inhibition of interfering stimuli and shifting attention from one task to another.

Despite the great amount of studies confirming this negative relationship, others could not find such effects or even revealed some in the opposite direction as summarized in the meta-analysis of Wiradhany and Nieuwenstein (2017). For example, the study of Ralph et al. (2014) found no significant relationships between media multitasking and attention switching or distractibility, while the study of Cardoso-Leite et al. (2016) found that individuals with intermediate levels of media multitasking even perform better in cognitive control than both light and heavy media multitaskers in some cases. Several studies even found evidence for completely opposing results, thus indicating that heavy media multitaskers were better able to switch between tasks (Alzahabi and Becker, 2013), and to employ a split mode of attention (Yap and Lim, 2013). To conclude, the meta-analysis of Wiradhany and Nieuwenstein (2017) considering all studies investigating media multitasking and cognitive control summarized that the often presumed association is most likely very small and therefore unlikely to be detected in studies employing small sample sizes. Therefore, according to them, the reason of this mixed set of results within this group might be the insufficient and greatly varying sample sizes or the inappropriateness of the proportional media multitasking measure used within studies so far.

### Topic Three: Media Multitasking, Learning and Academic Performance

This topic specifically bundles research on the impact of media multitasking on reading comprehension and academic performance. Since media multitasking is generally regarded to negatively affect human's cognitive capacity due to its complexity, it evidently raises some concerns related to people's reading, learning and academic performances. A detailed content analysis could distinguish two sub streams within this group related to the learning environment and more specifically to whether the studies investigated academic performance at home or at school. Regarding the home context, television and music appeared to be the two most common background media activities while students are reading, learning or doing homework. As such, various studies with divergent outcomes have been devoted to testing whether background television or music interferes with students' studying outcomes or not. The majority of these studies indicated that television watching during reading, learning, or doing homework is negatively related to students' performances, such as a cued-recall performance of an expository prose passage (Armstrong et al., 1991), information encoding performance of newspaper science articles (Armstrong and Chung, 2000), reading comprehension (Furnham et al., 1994) and homework performance (Pool et al., 2000). However, other research like the study of Cool et al. (1994), for example, found that there were no significant distractor effects of radio and television use on students' time spent studying, computational accuracy, reading comprehension, and reading rate. Furthermore, the study of Beentjes et al. (1996) pointed out that students' performance on paper-and-pencil assignments even somewhat increased by the use of background audio media and music television. With regard to the school context, the proliferation and ease of access to information and communication technologies, such as instant messaging, text messaging or Facebook, has been shown to increase students' tendency to engage in media multitasking during lectures and classes (Kraushaar and Novak, 2010). The prevalence of media multitasking at school and the cognitive depletion that comes with this behavior as explained before, obviously raises some concerns about students' academic performances. Indeed, various kinds of media multitasking behaviors in class, such as text messaging (Clayson and Haley, 2013), social media usage (Lau, 2017), mobile phone usage (Kuznekoff and Titsworth, 2013), and laptop usage (Fried, 2008; Sana et al., 2013; Gaudreau et al., 2014), have been demonstrated to be related to poor learning performances, lower course grades, and performance on tests.

### Topic Four: Media Multitasking and Information Consumption

The increased prevalence of simultaneous media usage among consumers evidently has some implications for media creators, media planners, and advertisers, as their content is now often viewed under divided attention. As people need to divide their processing resources among different media content streams, the resources they may allocate to one particular stream is limited by the occupation of some of their resources by the other task. As a result, a growing body of academic research addressed this topic and investigated the impact of media multitasking behaviors on the processing and outcomes of media and embedded persuasive content. While a small part of the research within this group focus on the effects of media multitasking behaviors on the processing, enjoyment and memory of media content (e.g., Kätsyri et al., 2016; Nee and Dozier, 2017; Rubenking, 2017), the majority of the studies investigate its implications for the processing and outcomes of persuasive and advertising messages (Jeong and Hwang, 2012; Segijn and Eisend, 2019).

The rapidly increasing amount of media multitasking research addressing advertising content was collected and investigated by two recent meta-analyses, which both seem to suggest that the direction of media multitasking effects are different for cognitive (i.e. attention, comprehension, and retention) compared to attitudinal advertising outcomes (i.e. persuasion, likeability and acceptance) and might be moderated by a multitude of additional factors (Jeong and Hwang, 2016; Segijn and Eisend, 2019). This is argued to be the case as a limited availability in cognitive resources does not only withhold people from storing, processing and memorizing advertising messages, but it also withholds them to critically process them, leading to better attitudinal outcomes (Jeong and Hwang, 2012; Segijn et al., 2016). As such, while various studies concluded that media multitasking has negative effects on consumers' advertising recognition, brand recognition, and brand memory (Duff and Sar, 2015; Angell et al., 2016), others revealed that this media consumption behavior has positive consequences for brand attitudes and purchase intention (Kazakova et al., 2016; Srivastava et al., 2016). However, just like the other topics, some inconsistent findings throughout the sampled studies can be found. As such, Segijn et al. (2017c) found no differences in brand memory between multi-screeners and single screeners when people have sufficient cognitive capacity. Besides, Segijn et al. (2016) even found negative effects of multiscreening (i.e. media multitasking with two screens) on consumers' affective advertising outcomes. Therefore, it is argued that characteristics of the specific media combinations and settings could serve as moderating variables and explain the diverging impact media multitasking behavior could have on advertising effects (Wang et al., 2015; Segijn and Eisend, 2019).

Within the literature so far, various moderators such as the relevance between two media activities (Van Cauwenberge et al., 2014; Segijn et al., 2017b), the integration of advertisement into a storyline (Yoon et al., 2011), advertising appeal types (Kazakova et al., 2016) and peoples' perceptual processing styles (Duff and Sar, 2015), have been detected to explain these mixed results. Furthermore, some researchers attempted to unravel the underlying mechanisms to better understand the effects of media multitasking on advertising effectiveness. For example, a recent meta-analysis conducted by Segijn and Eisend (2019) showed that attention allocation, perceived enjoyment and resistance to persuasive messages serve as driving mechanisms, explaining the impact of multiscreening on advertising memory and persuasion. To conclude, another substream of research can be identified within this group which specifically focuses on the impact of media multitasking on attitudes toward and the persuasiveness of political media content (e.g., Ran et al., 2016; Gottfried et al., 2017; Liu et al., 2020).

### Topic Five: Media Multitasking and Socioemotional Functions

As the media multitasking tendency strongly increased over the past decade, concerns about the negative consequences of this media consumption behavior for socioemotional functioning gave rise to a new stream of media multitasking research. As a broad concept, socioemotional functions consist of many components, among which emotion regulation, social success, psychological well-being, and sleep quality. Researchers have put forward two potential explanations for why media multitasking has a negative effect on socioemotional functioning (van Der Schuur et al., 2015). The first is based on the reasoning that media multitasking leads to deficits in cognitive control, which implies that people do not possess sufficient cognitive capacity to activate and regulate emotions (Becker et al., 2013). Thus, human socioemotional functions may be negatively affected when media multitasking due to cognitive depletion. The second explanation is based on the assumption that media multitaskers are more likely to use media during real-life interactions with others, which will decrease the quality of their face-to-face communication. As these face-to-face interactions with peers are recognized as key determinants of socioemotional development, the decreased quality of these may thus have considerable consequences (Pea et al., 2012). This was indeed confirmed by prior studies, which revealed a negative impact of media multitasking on socioemotional functions. For example, Becker et al. (2013) found that an increase in media multitasking tendency was associated with greater feelings of depression and social anxiety. Similarly, Pea et al. (2012) and Yang et al. (2015) found that media multitasking was associated with negative psychological well-being, indicated by constructs such as social success, feelings of normalcy, and self-evaluation. Besides, prior studies also suggested that media multitasking reduces people's sleep duration and pattern (Calamaro et al., 2009), which leads to more subsequent sleep problems such as fatigue, shortness of sleep, and loss of energy (van der Schuur et al., 2018).

Although most of the research until date supports the notion that media multitasking has a negative effect on socioemotional functioning, a study conducted by Shih (2013) showed a null relationship between media multitasking and well-being. Furthermore, the study of Xu et al. (2016) argued that the negative effects of media multitasking on socioemotional functions depend on the communication contexts. They found that media multitasking has no effect on social success during asynchronous social interactions such as emailing, texting, and online chatting. However, contrarily to the majority of research discussed above, they even found a positive effect of media multitasking on social success, normalcy and self-control during entertainment-driven media activities (e.g., watching video content, listening to music, playing video games). In addition, several studies found that media multitasking is positively correlated with perceived time passage (Chinchanachokchai et al., 2015; Xu and David, 2018).

## Interrelations Between Research Topics and Evolution Throughout the Years

As stated before, the topics of some media multitasking studies are manifold in character and could thus be categorized into multiple research topics. To quantify the relations among the different topics, we conducted a joint content analysis. The results showed that there were a total of 31 relations among groups, which involve 25 papers categorized into two topics and two papers categorized into three topics (see Appendix A). In particular, the first three topics appear to be strongly related to one another, while topic 4 and 5 are more independent. The analysis shows, for instance, that papers focusing on media multitasking in academic contexts also often investigate the fundamental issues of media multitasking with regard to cognitive outcomes.

**Figure 4:**
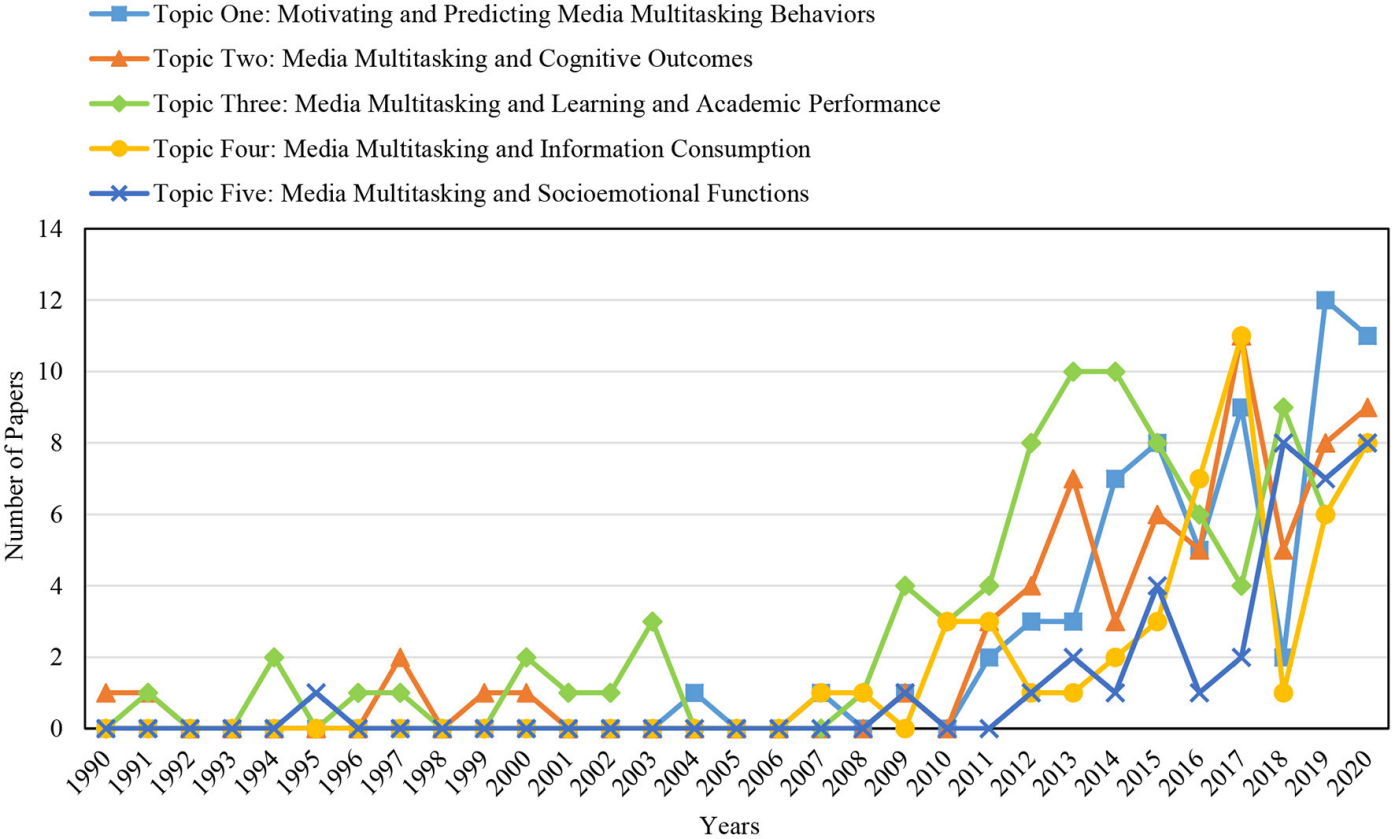
The evolution of research groups over years.

To better understand the evolution of media multitasking research over time, a dynamic content analysis was conducted for the papers within all topics independently. Figure 4 presents the number of papers within each research topic throughout the years. We can infer that the first three topics have been addressed ever since the rise of media multitasking research. This implies that media multitasking first tried grasping this complexity of media multitasking behavior by studying its prevalence, appearance, and predictors. Early research also showed interest in how this fairly new behavior at the time affected people's cognitive capacity and behavior, and what the consequences were for youngsters' reading comprehension and academic performance. It was not until 2007 that the implications of media multitasking behavior for the processing of media- and persuasive content (topic four) received attention. After that, a new research topic related to effects of media multitasking on socioemotional functions (topic five) gained research attention in 2009 and later years. Overall, the research within all groups has known a rapid growth in the past decade, which is consistent with the findings in the descriptive analyses.

## Research Gaps Within and Between the Different Topics

Even though research on media multitasking has greatly expanded over the past few years, the current approach helped us identifying several underexposed topics and shortcomings within the field.

Firstly, our analyses revealed a strong focus on adolescents within media multitasking research, by identifying the frequent occurrence of “adolescents,” “college students,” and “academic performance” as words/phrases in the title or keyword fields. This is in line with the popular assumption that especially youngsters are regular media multitaskers. However, this common belief has been disproved by the extensive diary research of Voorveld and van der Goot (2013), arguing that media multitasking behavior is common among all ages. This highlights an important research gap in the field of media multitasking, as younger and older media multitaskers received little to no research attention so far.

Secondly, regardless of the research topics, the majority of media multitasking research explains their effects based on the assumption of cognitive depletion (e.g., Jeong and Hwang, 2012; Wei et al., 2012). Even though some researchers started to address other, more affective underlying mechanisms of media multitasking effects such as enjoyment and time perception (e.g., Chinchanachokchai et al., 2015; Park et al., 2019), we still identified a dominant focus on the driving role of cognitive depletion within all research topics. This focus is most certainly the result of the greatly influential paper of Ophir et al. (2009), which firstly associated media multitasking frequency with cognitive control. This particular paper received the most local and global citation scores and the highest altmetric score within our sample. This indicates that their paper was most cited within and without the field of media multitasking and was often discussed and shared online. As a result, the research within the identified clusters has been focusing on the consequent effects of media multitasking and cognitive depletion for academic performance, the processing of media content, and socioemotional outcomes. However, it must be noted that previous research outside the field of media multitasking has been revealing that cognitive control is necessary to protect oneself against a great amount of other dependent undesired effects as well. For example, a lack in cognitive control is argued to be risk factor for a broad range of undesired behavioral and impulse-control outcomes such as binge eating, unhealthy food choices, alcohol, and drug use (e.g., Heatherton and Baumeister, 1991; Baumeister et al., 2007; Friese et al., 2008), which are all topics that have not been addressed in media multitasking research so far. To conclude, cognitive depletion has been predominantly measured by cognitive performance tasks such as the Stroop task (Stroop, 1992), for example, while implicit measurement methods such as functional magnetic resonance imaging (fMRI) and electroencephalography (EEG) were only used within very few studies (e.g., Loh and Kanai, 2014; Moisala et al., 2016).

Thirdly, it must be noted that the interrelation analysis between the different research topics clearly indicated that some research topics are more commonly combined while other topics do not often co-occur together in one paper. As such, the two topics that were most frequently combined within media multitasking research were cognitive control (cf. topic two) and academic performance (cf. topic three). Besides, while the studies within the fourth and fifth topic often used depletion theories to build up their conceptual framework, they did not often actually measure depletion. More specifically, while they do touch these topics in their theoretical frameworks, only the very few researches that actually test the underlying role of this underlying mechanism (e.g., Beuckels et al., 2019 who measured cognitive load as mediator) were categorized in research topic two as well. Therefore, in contrast with the dominant focus on cognitive theories such as the limited capacity model (Lang, 2000) to explain effects in cluster four and five, these research topics were not often interrelated to research topic two. Other topics that were rarely addressed together in papers but that are suggested to be related to each other are cluster three and five. More specifically, one paper that specifically combined these topics suggests that the relationship between academic performance and well-being is reciprocal among today's media multitasking youth (Luo et al., 2020). However, more research simultaneously addressing the subjects of these two topics is non-existing until date.

As a fourth point, the high appearance of the keyword “television” suggests that this medium has been most frequently investigated by prior media multitasking research. However, it is plausible to expect that this predominant focus on television in media multitasking research is a result of the earlier introduction of this medium compared to others such as internet and mobile phone use. As the popularity of social network sites (SNSs) and the mobile phone use only really took off over the past 10 years, publications about these media in the multitasking literature only made an entrance in recent years. This was translated into the fact that keywords such as mobile phone and Facebook only appeared in more recent papers and show to increasingly gain popularity. In conclusion, while recent reports argue that 72% of adolescents report to media multitask with television and social network sites nowadays (GlobalWebIndex, 2019), this is not completely translated into the field of media multitasking research so far. While some studies started to show interest by investigating specific media multitasking behavior with social network sites (e.g., Beuckels et al., 2017; Weimann-Saks et al., 2020), this particular media multitasking behavior is not yet accounted for within all research topics until date.

To conclude, the current bibliometric study could be grouped into 11 clusters and five research topics in total. While these groups bundle the most frequently addressed topics within media multitasking research so far, we noticed the rise of some specific new topics within the past few years. More specifically, we noticed that six studies within the last years specifically focused on media multitasking behavior when watching sport events on television. This specific focus on media multitasking while watching sports broadcastings did not receive any attention before 2018 (Jensen et al., 2018), while the other five papers on this topic have only been published in 2020 (e.g., Billings et al., 2020; Tamir, 2020). As the interest in this topic has thus only emerged within the last years, it is evident that they did not yet initiate a full-fledged research topic. In general, past studies did not yet focused in detail on the thematic type of content consumed in the media multitasking context. Besides, while previous research often expressed concerns related to the mental health of media multitaskers, it is only in recent years that academics showed interest in the impact of media multitasking behavior on individuals' physical health. This was translated in a few studies investigating whether media multitasking affects food consumption patterns (e.g., Marsh et al., 2015; Lopez et al., 2019) and one study specifically investigating whether ones' media multitasking tendency would affect his health-protective behavioral intentions (Kononova et al., 2017).

## Directions for Future Research

The foregoing analyses and the consequently identified research gaps point toward several key topics and areas which provide some highly interesting starting points for future media multitasking research. The current section will highlight the issues of which we believe are the most compelling for future investigation.

### Methodological Issues

As discussed above, most media multitasking studies use one or another version of the ‘*limited capacity model of mediated message processing*’ (Lang, 2000) within their theoretical framework. Besides, while many studies in research topic two and three implicitly measured cognitive outcomes such as task switching, working memory capacity and filtering of information with performance tasks or eye-tracking devices, these constructs were much less accounted for within the more applied studies from topic four and five. A possible explanation for this finding might be that the studies in these latter topics often rely on self-reported measures through questionnaires, which are difficult to assess after making participants engage in performance tasks. That is because it would be impossible to determine whether the effects on the self-reported outcomes would be due to the cognitive depletion while media multitasking or while performing the cognitive performance tasks just before filling out the questionnaire. While the broad field of (general) multitasking studies greatly employs neuropsychological measurement methods such as EEG or fMRI, only few media multitasking studies adopted these methods so far (Moisala et al., 2016). Therefore, it would be interesting for future media multitasking studies to equally measure cognitive resource depletion in implicit ways, as this would allow them to unambiguously link cognitive depletion to the self-reported dependent variables from topic four and five (e.g., advertising effectiveness or emotional well-being).

Additionally, media multitasking frequency as widely measured by the Media Multitasking Index (MMI; originally designed by Ophir and colleagues and adopted by nearly all studies within research topic two) indicates how often one multitasks when using media. As such, a person who only uses media for 30 min per day but media multitasks 90% of that time is considered as a greater media multitaskers than a person who engages in 12 h of media use per day but media multitasks “only” 50% of that time. Therefore, it might be interesting for future research to consider other media multitasking measures and larger sample sizes when further exploring the association between media multitasking and cognitive control.

### Target Groups

As argued above, our analyses revealed that media multitasking research knows a strong focus on adolescents. However, as it appears that people across all ages are fervent media multitaskers nowadays (Voorveld and van der Goot, 2013), it might be opportune for future research to start focusing on individuals from other age groups within their research. Especially because the majority of media multitasking research assumes negative outcomes due to the situational decrease in cognitive control when media multitasking, it might be particularly interesting to investigate how young children cope with certain media situations. More specifically, neuropsychological research argued that children are more easily distracted by irrelevant stimuli compared to adults due to their immature frontal lobe contributions and thus lower levels of behavioral control (Bunge et al., 2002).

Therefore, future research could investigate whether during media multitasking children do or do not experience stronger detrimental effects compared to media multitaskers of an older age. Since a large amount of children has their own mobile device nowadays (Kabali et al., 2015), it might be expected that mobile devices are also increasingly finding their ways into elementary or college classes nowadays. However, nearly all studies on the impact of media multitasking on academic performance has been performed among adolescents (cf. research topic three). Therefore, it might be interesting for scholars to broaden the scope of this research topic by involving other age groups. Previous research also indicates that media-users from older generations report more difficulties when media multitasking compared to those from younger generations. As enjoyment appears to be an important driver of media multitasking effects (e.g., Chinchanachokchai et al., 2015), it might be interesting for future research to address whether these encountered difficulties among older age groups would affect media multitasking outcomes through reduced levels of enjoyment.

### Explanatory Processes

Many studies within our sample have been shown that media multitasking can lead to a temporal reduction in one's cognitive control and that this consequently affects, for example, how people perform at school (e.g., Wei et al., 2012), react to persuasive media content (e.g., Jeong and Hwang, 2012) and how they feel (Wiradhany and Koerts, 2019). While these are very important findings, cognitive research argues that executive control is equally indispensable to bolster ourselves against a wide range of other undesired effects in daily life. More specifically, as it is argued that prior behaviors that require high intensity in cognitive control could decrease the performance on subsequent tasks that also require cognitive control resources (e.g., Muraven and Baumeister, 2000; Hofmann et al., 2012), it might be particularly interesting for future research to investigate how engaging in media multitasking affects people's reactions to unhealthy habits, gambling and other behaviors that require self-regulation.

Further, it might be interesting to examine how media multitasking affects people's processing of the media content. As suggested by the shallowing hypothesis, a frequent use of digital media fosters shallow thought and decreases people's tendency to use reflective thought (Annisette and Lafreniere, 2017). This way of thinking is often induced by time constraints as studies have shown lower reading comprehension and less successful problem solving on screen when participants faced time pressure (e.g., Sidi et al., 2017; Delgado and Salmerón, 2021). In addition, people who often use social media tend to prefer morally shallow life goals such as hedonism and image over goals related to morality and aesthetics. It would be interesting whether media multitasking induces superficial processing of the content and whether these shallow thought processes can explain the effects of media multitasking behavior.

Other potential mediating variables explaining media multitasking effects, besides cognitive control and visual attention, remain greatly underexposed within the field of media multitasking research (e.g., the perceived feeling of control, fear of missing out, perceived ability to process all information). Therefore, future research should aim to transcend the great overreliance on the cognitive mediators, as this one-sided approach might withhold us to get a complete picture of the media multitasking story.

### Media Types

The results of the current study revealed that media multitasking studies do not only have a dominant focus on a certain audience and mediating variables, but also greatly focused on television watching when media multitasking. Even though the television has been widely adopted and integrated in current households, today's media landscape is rapidly evolving which urges for more research on popular media multitasking combinations in recent years. As such, while today's youth is exhaustively engaging in media multitasking with SNSs nowadays (Reinecke et al., 2017; GlobalWebIndex, 2019), only few media multitasking studies investigated the consequences of this particular media consumption behavior so far (e.g., Rosen et al., 2013). Within the context of advertising research, it has been argued that the great amount of social content on these SNSs negatively affects people's state self-esteem, whereby it induces different processes compared to media multitasking with non-social media (Beuckels et al., 2017). Especially because research has been shown that decreases in self-esteem make people rely more strongly on the opinion of others (Bither and Wright, 1973), it might be expected that media multitasking with SNSs would affect media-users socioemotional well-being and could make them susceptible toward persuasive attempts of others. Many studies also indicated that social media use correlates to depressive feelings (Frison and Eggermont, 2016) and might induce an addiction or dependency to the SNSs (especially among adolescents). Research examining the influence of social connectedness during media multitasking might influence this type of media experience. However, the complete subject of so called “social media multitasking” remains fairly underexplored until date, offering a broad and interesting opportunity for future research.

### Media Content

As shortly discussed in the “research gap” section, some very specific research topics have emerged over the past few years and deserve some further investigation within the future. As such, we noticed that especially within the last year, a strong increasing interest in media multitasking behavior when watching sports can be witnessed (e.g., Billings et al., 2020). As such, it is very common nowadays for sport spectators to watch live broadcasting sports events, while simultaneously engaging in online platforms for more sports content or to interact with other viewers (Sezen et al., 2020). More specifically, the study of Rubenking (2017) revealed that using second screens to interact with content related to the sport event could have a great positive effect on the perceived enjoyment among media-users. It might be interesting for future research to further investigate further consequences of this particular media multitasking behavior and the associated pleasure. At the same time, focusing on pleasure and enjoyment as driving mechanisms of media multitasking effects when watching specific media content would immediately tackle the above-mentioned overreliance on the cognitive depletion perspective as well.

As a final point, we would suggest for future research to further dig into the effect of media multitasking on (un)healthy behavior. For example, it has been suggested that both the presence of screens and the amount of distraction independently encourage people to eat more (e.g., Hetherington et al., 2006; Jackson et al., 2009). As media multitasking contexts are typically characterized with both screens and distractions, it might thus be particularly interesting to investigate how this behavior affects people's food intake. This issue has already been tackled by few studies (e.g., Lopez et al., 2019) but scholars call for further (longitudinal) research to tackle, among others, the question of causality between media multitasking and higher Body Mass Index. Besides, as it has been shown that media multitaskers are less capable of counterarguing persuasive messages due to cognitive constraints (e.g., Jeong and Hwang, 2012), future research could also investigate whether advertisements promoting healthy food behavior could also benefit from this media consumption behavior.

## Conclusion

To conclude, this study provides an insight into the research on media multitasking in order to provide guidance for future research. The juggling with different media tasks has become an omnipresent media behavior among all ages and all types of consumers. Accelerated by technological evolutions and the rapid emergence of new content types (streaming of television, rise of social media, etc.), media multitasking has affected the lives of many. Current research on media multitasking can be classified into five main topics, focusing on people's motivations to media multitask, the cognitive deficits related to this behavior and the impacts it has on three different outcomes: learning and academic performance; processing of media and advertising content; and socioemotional well-being. Insights from these studies helped us identifying research gaps. Accordingly, a future research agenda in terms of methodological issues, target groups, explanatory processes, media types and media content was proposed, and we hope that this may guide future research on media multitasking.

## Data Availability Statement

The original contributions presented in the study are included in the article/Supplementary Material, further inquiries can be directed to the corresponding author/s.

## Author Contributions

EB contributed to the original conceptualization of the study, developed the theory, and wrote the original draft of the theoretical part of the paper. GY performed the analytic calculations and wrote the first draft of the methodological part of the paper. LH contributed to the original conceptualization of the study and assisted in the process of writing, reviewing, and editing the paper. VC assisted in the process of writing, reviewing, and editing the paper. All authors contributed to the article and approved the submitted version.

## Conflict of Interest

The authors declare that the research was conducted in the absence of any commercial or financial relationships that could be construed as a potential conflict of interest.
