# Supplementary material for: Media Multitasking: A Bibliometric Approach and Literature Review
Source: Front Psychol. 2021 Jun 23;12:623643. doi: 10.3389/fpsyg.2021.623643 (PMC8260967; doi:10.3389/fpsyg.2021.623643)
Supplement: Supplementary file 1 [file Table_1.DOCX]

Supplementary Material

# Supplementary Tables

Appendix A The paper list

|  | Author(s) | Title | Year | Topic 1 | Topic 2 | Topic 3 | Topic 4 | Topic 5 |
| --- | --- | --- | --- | --- | --- | --- | --- | --- |
| 1 | Armstrong G. B.; Greenberg B. S. | Background television as an inhibitor of cognitive processing | 1990 |  | √ |  |  |  |
| 2 | Armstrong G. B.; Boiarsky G. A.; Mares M. L. | Background television and reading performance | 1991 |  | √ | √ |  |  |
| 3 | Furnham A.; Gunter B.; Peterson, E. | Television distraction and the performance of introverts and extroverts | 1994 |  |  | √ |  |  |
| 4 | Cool, V. A.; Yarbrough, D. B.; Patton, J. E.; Runde, R.; Keith, T. Z. | Experimental effects of radio and television distractors on children’s performance on mathematics and reading assignments | 1994 |  |  | √ |  |  |
| 5 | Oldham, G. R.; Cummings, A.; Mischel, L. J.; Schmidtke, J. M.; Zhou, J. | Listen while you work? Quasi-experimental relations between personal-stereo headset use and employee work responses | 1995 |  |  |  |  | √ |
| 6 | Beentjes, J. W.; Koolstra, C. M.; Van der Voort, T. H. | Combining background media with doing homework: Incidence of background media use and perceived effects | 1996 |  |  | √ |  |  |
| 7 | Armstrong G. B.; Sopory P. | Effects of background television on phonological and visuo-spatial working memory | 1997 |  | √ |  |  |  |
| 8 | Furnham A.; Bradley A. | Music while you work: The differential distraction of background music on the cognitive test performance of introverts and extraverts. | 1997 |  | √ | √ |  |  |
| 9 | Beh, H. C.; Hirst, R. | Performance on driving-related tasks during music | 1999 |  | √ |  |  |  |
| 10 | Pool M. M.; Van Der Voort T. H.; Beentjes J. W.; Koolstra C. M. | Background television as an inhibitor of performance on easy and difficult homework assignments | 2000 |  |  | √ |  |  |
| 11 | Armstrong G. B.; Chung L. | Background television and reading memory in context: Assessing TV interference and facilitate context effects on encoding versus retrieval processes | 2000 |  | √ | √ |  |  |
| 12 | Ransdell, S. E.; Gilroy, L. | The effects of background music on word processed writing | 2001 |  |  | √ |  |  |
| 13 | Kallinen, K. | Reading news from a pocket computer in a distracting environment: Effects of the tempo of background music | 2002 |  |  | √ |  |  |
| 14 | Pool M. M.; Koolstra C. M.; Van der Voort T. H. | Distraction effects of background soap operas on homework performance: An experimental study enriched with observational data | 2003 |  |  | √ |  |  |
| 15 | Pool M. M.; Koolstra C. M.; van der Voor, T. H. A. | The impact of background radio and television on high school students’ homework performance | 2003 |  |  | √ |  |  |
| 16 | Hembrooke, H.; Gay, G. | The laptop and the lecture: The effects of multitasking in learning environments | 2003 |  |  | √ |  |  |
| 17 | Pilotta J. J.; Schultz D. E.; Drenik G.; Rist P. | Simultaneous media usage: A critical consumer orientation to media planning | 2004 | √ |  |  |  |  |
| 18 | Jeong, S. H.; Fishbein, M. | Predictors of multitasking with media: Media factors and audience factors | 2007 | √ |  |  |  |  |
| 19 | Chowdhury R. M.; Finn A.; Olsen G. D. | Investigating the simultaneous presentation of advertising and television programming | 2007 |  |  |  | √ |  |
| 20 | Fried, C. B. | In-class laptop use and its effects on student learning | 2008 |  |  | √ |  |  |
| 21 | Collins R. L. | Media multitasking: Issues posed in measuring the effects of television sexual content exposure | 2008 |  |  |  | √ |  |
| 22 | Carrier, L. M.; Cheever, N. A.; Rosen, L. D.; Benitez, S.; Chang, J. | Multitasking across generations: Multitasking choices and difficulty ratings in three generations of Americans | 2009 | √ |  |  |  |  |
| 23 | Ophir E.; Nass C.; Wagner A. D. | Cognitive control in media multitaskers | 2009 |  | √ |  |  |  |
| 24 | Lin L.; Robertson T.; Lee J. | Reading performances between novices and experts in different media multitasking environments | 2009 |  |  | √ |  |  |
| 25 | Fox, A. B.; Rosen, J.; Crawford, M. | Distractions, distractions: Does instant messaging affect college students’ performance on a concurrent reading comprehension task? | 2009 |  |  | √ |  |  |
| 26 | End, C. M.; Worthman, S.; Mathews, M. B.; Wetterau, K. | Costly cell phones: The impact of cell phone rings on academic performance | 2009 |  |  | √ |  |  |
| 27 | Benbunan-Fich, R.; Truman, G. E. | Technical opinion multitasking with laptops during meetings | 2009 |  |  | √ |  |  |
| 28 | Calamaro, C. J.; Mason, T. B.; Ratcliffe, S. J. | Adolescents living the 24/7 lifestyle: Effects of caffeine and technology on sleep duration and daytime functioning | 2009 |  |  |  |  | √ |
| 29 | Bowman L. L.; Levine L. E.; Waite B. M.; Gendron M. | Can students really multitask? An experimental study of instant messaging while reading | 2010 |  |  | √ |  |  |
| 30 | Ellis, Y.; Daniels, B.; Jauregui, A. | The effect of multitasking on the grade performance of business students | 2010 |  |  | √ |  |  |
| 31 | Kraushaar, J. M.; Novak, D. C. | Examining the affects of student multitasking with laptops during the lecture | 2010 |  |  | √ |  |  |
| 32 | Bardhi F.; Rohm A. J.; Sultan F. | Tuning in and tuning out: Media multitasking among young consumers | 2010 |  |  |  | √ |  |
| 33 | Zhang W.; Jeong S. H.; Fishbein M. | Situational factors competing for attention: The interaction effect between multitasking and sexual explicitness on TV recognition | 2010 |  |  |  | √ |  |
| 34 | Jeong S. H.; Hwang Y.; Fishbein M. | Effects of exposure to sexual content in the media on adolescent sexual behaviors: The moderating role of multitasking with media | 2010 |  |  |  | √ |  |
| 35 | Zhong B.; Hardin M.; Sun T. | Less effortful thinking leads to more social networking? The associations between the use of social network sites and personality traits | 2011 | √ |  |  |  |  |
| 36 | Judd, T.; Kennedy, G. | Measurement and evidence of computer-based task switching and multitasking by ‘Net Generation’ students | 2011 | √ |  |  |  |  |
| 37 | Kämpfe, J.; Sedlmeier, P.; Renkewitz, F. | The impact of background music on adult listeners: A meta-analysis | 2011 |  | √ | √ | √ |  |
| 38 | Brasel S. A.; Gips J. | Media multitasking behavior: Concurrent television and computer usage | 2011 |  | √ |  |  |  |
| 39 | Cain M. S.; Mitroff S. R. | Distractor filtering in media multitaskers | 2011 |  | √ |  |  |  |
| 40 | Junco R.; Cotten S. R. | Perceived academic effects of instant messaging use | 2011 |  |  | √ |  |  |
| 41 | Lin, L.; Lee, J.; Robertson, T. | Reading while watching video: The effect of video content on reading comprehension and media multitasking ability | 2011 |  |  | √ |  |  |
| 42 | Rosen, L. D.; Lim, A. F.; Carrier, L. M.; Cheever, N. A. | An empirical examination of the educational impact of text message-induced task switching in the classroom: Educational implications and strategies to enhance learning | 2011 |  |  | √ |  |  |
| 43 | Voorveld H. A. M. | Media multitasking and the effectiveness of combining online and radio advertising | 2011 |  |  |  | √ |  |
| 44 | Yoon, S.; Choi, Y. K.; Song, S. | When intrusive can be likable | 2011 |  |  |  | √ |  |
| 45 | Ie A.; Haller C. S.; Langer E. J.; Courvoisier D. S. | Mindful multitasking: The relationship between mindful flexibility and media multitasking | 2012 | √ |  |  |  |  |
| 46 | Wang Z.; Tchernev J. M. | The “myth” of media multitasking: Reciprocal dynamics of media multitasking, personal needs, and gratifications | 2012 | √ |  |  |  |  |
| 47 | Zhang, W.; Zhang, L. | Explicating multitasking with computers: Gratifications and situations | 2012 | √ |  |  |  |  |
| 48 | Lui K. F. H.; Wong A. C. N. | Does media multitasking always hurt? A positive correlation between multitasking and multisensory integration | 2012 |  | √ |  |  |  |
| 49 | Wang, Z.; David, P.; Srivastava, J.; Powers, S.; Brady, C.; D’Angelo, J.; Moreland, J. | Behavioral performance and visual attention in communication multitasking: A comparison between instant messaging and online voice chat | 2012 |  | √ |  |  |  |
| 50 | Lee J.; Lin L.; Robertson T. | The impact of media multitasking on learning | 2012 |  |  | √ |  |  |
| 51 | Junco, R.; Cotten, S. R. | No A 4 U: The relationship between multitasking and academic performance | 2012 |  |  | √ |  |  |
| 52 | Junco, R. | In-class multitasking and academic performance | 2012 |  |  | √ |  |  |
| 53 | Wei, F. Y. F.; Wang, Y. K.; Klausner, M. | Rethinking college students’ self-regulation and sustained attention: Does text messaging during class influence cognitive learning? | 2012 |  | √ | √ |  |  |
| 54 | Wood, E.; Zivcakova, L.; Gentile, P.; Archer, K.; De Pasquale, D.; Nosko, A. | Examining the impact of off-task multi-tasking with technology on real-time classroom learning | 2012 |  |  | √ |  |  |
| 55 | Douglas, D.; Angel, H.; Bethany, W. | Digital devices, distraction, and student performance: Does in-class cell phone use reduce learning? | 2012 |  |  | √ |  |  |
| 56 | Burak, L. J. | Multitasking in the university classroom | 2012 |  |  | √ |  |  |
| 57 | Jeong, S. H.; Hwang Y. | Does multitasking increase or decrease persuasion? Effects of multitasking on comprehension and counterarguing | 2012 |  |  |  | √ |  |
| 58 | Levine L. E.; Waite B. M.; Bowman L. L. | Mobile media use, multitasking and distractibility | 2012 |  | √ | √ |  |  |
| 59 | Pea R.; Nass C.; Meheula L.; Rance M.; Kumar A.; Bamford H.; Nass M.; Simha A.; Stillerman B.; Yang S.; Zhou M. | Media use, face-to-face communication, media multitasking, and social well-being among 8- to 12-year-old girls | 2012 |  |  |  |  | √ |
| 60 | David P.; Xu L.; Srivastava J.; Kim J. H. | Media multitasking between two conversational tasks | 2013 |  | √ |  |  |  |
| 61 | Sanbonmatsu D. M.; Strayer D. L.; Medeiros-Ward N.; Watson J. M. | Who multi-tasks and why? Multi-tasking ability, perceived multi-tasking ability, impulsivity, and sensation seeking | 2013 | √ | √ |  |  |  |
| 62 | Voorveld H. A. M.; van der Goot M. | Age differences in media multitasking: A diary study | 2013 | √ |  |  |  |  |
| 63 | Lin L. | Multiple dimensions of multitasking phenomenon | 2013 |  | √ |  |  |  |
| 64 | Kononova A. | Multitasking across borders: A cross-national study of media multitasking behaviors, its antecedents, and outcomes | 2013 | √ | √ |  |  |  |
| 65 | Minear M.; Brasher F.; McCurdy M.; Lewis J.; Younggren A. | Working memory, fluid intelligence, and impulsiveness in heavy media multitaskers | 2013 |  | √ |  |  |  |
| 66 | Yap J. Y.; Lim S. W. H. | Media multitasking predicts unitary versus splitting visual focal attention | 2013 |  | √ |  |  |  |
| 67 | Alzahabi R.; Becker M. W. | The association between media multitasking, task-switching, and dual-task performance | 2013 |  | √ |  |  |  |
| 68 | Song K. S.; Nam S. C.; Lim H.; Kim J. K. | Analysis of youngsters’ media multitasking behaviors and effect on learning | 2013 |  |  | √ |  |  |
| 69 | Clayson, D. E.; Haley, D. A. | An introduction to multitasking and texting: Prevalence and impact on grades and GPA in marketing classes | 2013 |  |  | √ |  |  |
| 70 | Sana, F.; Weston, T.; Cepeda, N. J. | Laptop multitasking hinders classroom learning for both users and nearby peers | 2013 |  |  | √ |  |  |
| 71 | Karpinski, A. C.; Kirschner, P. A.; Ozer, I.; Mellott, J. A.; Ochwo, P. | An exploration of social networking site use, multitasking, and academic performance among United States and European university students | 2013 |  |  | √ |  |  |
| 72 | kuznekoff, J. H.; Titsworth, S. | The impact of mobile phone usage on student learning | 2013 |  |  | √ |  |  |
| 73 | Rosen, L. D.; Carrier, L. M.; Cheever, N. A | Facebook and texting made me do it: Media-induced task-switching while studying | 2013 |  |  | √ |  |  |
| 74 | Subrahmanyam, K.; Michikyan, M.; Clemmons, C.; Carrillo, R.; Uhls, Y. T.; Greenfield, P. M. | Learning from paper, learning from screens: Impact of screen reading and multitasking conditions on reading and writing among college students | 2013 |  |  | √ |  |  |
| 75 | Fante, R.; Jacobi, L. L.; Sexton, V. D. | The effects of instant messaging and task difficulty on reading comprehension | 2013 |  |  | √ |  |  |
| 76 | Tran, P.; Carrillo, R.; Subrahmanyam, K. | Effects of online multitasking on reading comprehension of expository text | 2013 |  |  | √ |  |  |
| 77 | McDonald, S. | The effects and predictor value of in-class texting behavior on final course grades | 2013 |  |  | √ |  |  |
| 78 | Srivastava J. | Media multitasking performance: Role of message relevance and formatting cues in online environments | 2013 |  |  |  | √ |  |
| 79 | Shih S. I. | A null relationship between media multitasking and well-being | 2013 |  |  |  |  | √ |
| 80 | Becker M. W.; Alzahabi R.; Hopwood C. J. | Media multitasking is associated with symptoms of depression and social anxiety | 2013 |  |  |  |  | √ |
| 81 | Voorveld H. A. M.; Segijn C. M.; Ketelaar P. E.; Smit E. G. | Investigating the prevalence and predictors of media multitasking across countries | 2014 | √ |  |  |  |  |
| 82 | Bowman L. L.; Waite B. M.; Levine L. E. | A cross-cultural comparison of media multitasking in American and Malaysian college students | 2014 | √ |  | √ |  |  |
| 83 | Kononova A.; Zasorina T.; Diveeva N.; Kokoeva A.; Chelokyan A. | Multitasking goes global: Multitasking with traditional and new electronic media and attention to media messages among college students in Kuwait, Russia, and the USA | 2014 | √ |  | √ |  |  |
| 84 | Cotten S. R.; Shank D. B.; Anderson W. A. | Gender, technology use and ownership, and media-based multitasking among middle school students | 2014 | √ |  |  |  |  |
| 85 | Hwang Y.; Kim H.; Jeong S. H | Why do media users multitask?: Motives for general, medium-specific, and content-specific types of multitasking | 2014 | √ |  |  |  |  |
| 86 | Duff, B. R. L.; Yoon, G.; Wang, Z.; Anghelcev, G. | Doing it all: An exploratory study of predictors of media multitasking | 2014 | √ |  |  |  |  |
| 87 | Loh K. K.; Kanai R. | Higher media multi-tasking activity is associated with smaller gray-matter density in the anterior cingulate cortex | 2014 |  | √ |  |  |  |
| 88 | Baumgartner S. E.; Weeda W. D.; van der Heijden L. L.; Huizinga M. | The relationship between media multitasking and executive function in early adolescents | 2014 |  | √ |  |  |  |
| 89 | Ralph B .C. W.; Thomson D. R.; Cheyne J. A.; Smilek D. | Media multitasking and failures of attention in everyday life | 2014 |  | √ |  |  |  |
| 90 | Calderwood C.; Ackerman P. L.; Conklin E. M. | What else do college students “do” while studying? An investigation of multitasking | 2014 |  |  | √ |  |  |
| 91 | Shah III A. V.; Mullens III D. J.; Van Duyn III L. J.; Januchowski R. P. | Multitasking behaviors of osteopathic medical students | 2014 |  |  | √ |  |  |
| 92 | Judd T. | Making sense of multitasking: The role of Facebook | 2014 | √ |  | √ |  |  |
| 93 | Dietz, S.; Henrich, C. | Texting as a distraction to learning in college students | 2014 |  |  | √ |  |  |
| 94 | Gaudreau, P.; Miranda, D.; Gareau, A. | Canadian university students in wireless classrooms: What do they do on their laptops and does it really matter? | 2014 |  |  | √ |  |  |
| 95 | Ravizza, S. M.; Hambrick, D. Z.; Fenn, K. M. | Non-academic internet use in the classroom is negatively related to classroom learning regardless of intellectual ability | 2014 |  |  | √ |  |  |
| 96 | Wei, F. Y. F.; Wang, Y. K.; Fass, W. | An experimental study of online chatting and notetaking techniques on college students’ cognitive learning from a lecture | 2014 |  |  | √ |  |  |
| 97 | Conard, M. A.; Marsh, R. F | Interest level improves learning but does not moderate the effects of interruptions: An experiment using simultaneous multitasking | 2014 |  |  | √ |  |  |
| 98 | Van Cauwenberge A.; Schaap G.; Van Roy R. | “TV no longer commands our full attention”: Effects of second-screen viewing and task relevance on cognitive load and learning from news | 2014 |  |  |  | √ |  |
| 99 | Gunawardena T.; Waiguny M. K | So many things to do! How multitasking affects memory and attitude for product and brand placements | 2014 |  |  |  | √ |  |
| 100 | Bowman, J. M.; Pace, R. C. | Dual-tasking effects on outcomes of mobile communication technologies | 2014 |  |  |  |  | √ |
| 101 | Aagaard J. | Media multitasking, attention, and distraction: A critical discussion | 2015 |  |  | √ |  |  |
| 102 | Kononova A.; Chiang Y. H. | Why do we multitask with media? Predictors of media multitasking among Internet users in the United States and Taiwan | 2015 | √ |  |  |  |  |
| 103 | Christensen C. G.; Bickham D.; Ross C. S.; Rich M. | Multitasking with television among adolescents | 2015 | √ |  |  |  |  |
| 104 | Voorveld H. A. M.; Viswanathan V. | An observational study on how situational factors influence media multitasking with TV: The role of genres, dayparts, and social viewing | 2015 | √ |  |  |  |  |
| 105 | Székely L. | The typology of multitasking activity | 2015 | √ |  |  |  |  |
| 106 | Cardoso-Leite P.; Green C. S.; Bavelier D. | On the impact of new technologies on multitasking | 2015 | √ |  |  |  |  |
| 107 | Gil de Zúñiga, H.; Garcia-Perdomo, V.; McGregor, S. C. | What is second screening? Exploring motivations of second screen use and its effect on online political participation | 2015 | √ |  |  |  |  |
| 108 | Lang, A.; Chrzan, J. | Media multitasking: Good, bad, or ugly? | 2015 | √ | √ |  |  |  |
| 109 | Vatavu R. D.; Mancas M. | Evaluating visual attention for multi-screen television: Measures, toolkit, and experimental findings | 2015 |  | √ |  |  |  |
| 110 | Kazakova S.; Cauberghe V.; Pandelaere M.; De Pelsmacker P. | Can’t see the forest for the trees? The effect of media multitasking on cognitive processing style | 2015 |  | √ |  |  |  |
| 111 | Ralph B. C. W.; Thomson D. R.; Seli P.; Carriere J. S. A.; Smilek D. | Media multitasking and behavioral measures of sustained attention | 2015 |  | √ |  |  |  |
| 112 | Wang Z.; Irwin M.; Cooper C.; Srivastava J. | Multi-dimensions of media multitasking and adaptive media selection | 2015 |  | √ |  |  |  |
| 113 | Bellur S.; Nowak K. L.; Hull K. S. | Make it our time: In class multitaskers have lower academic performance | 2015 |  |  | √ |  |  |
| 114 | Mokhtari K.; Delello J.; Reichard C. | Connected yet distracted: Multitasking among college students | 2015 |  |  | √ |  |  |
| 115 | Hassoun D. | “All over the place”: A case study of classroom multitasking and attentional performance | 2015 |  |  | √ |  |  |
| 116 | Carrier L. M.; Rosen L. D.; Cheever N. A.; Lim A. F. | Causes, effects, and practicalities of everyday multitasking | 2015 | √ |  | √ |  |  |
| 117 | Zhang, W. | Learning variables, in-class laptop multitasking and academic performance: A path analysis. | 2015 |  |  | √ |  |  |
| 118 | Lawson, D.; Henderson, B. B. | The costs of texting in the classroom | 2015 |  |  | √ |  |  |
| 119 | Duff B. R. L.; Sar S. | Seeing the big picture: Multitasking and perceptual processing influences on ad recognition | 2015 |  |  |  | √ |  |
| 120 | Chinchanachokchai S.; Duff B. R. L.; Sar S. | The effect of multitasking on time perception, enjoyment, and ad evaluation | 2015 |  |  |  | √ |  |
| 121 | Jeong, S. H.; Hwang, Y. | Multitasking and persuasion: The role of structural interference | 2015 |  |  |  | √ |  |
| 122 | Van Der Schuur W. A.; Baumgartner S. E.; Sumter S. R.; Valkenburg P. M. | The consequences of media multitasking for youth: A review | 2015 |  | √ | √ |  | √ |
| 123 | Yang X.; Xu X.; Zhu L. | Media multitasking and psychological wellbeing in Chinese adolescents: Time management as a moderator | 2015 |  |  |  |  | √ |
| 124 | Marsh S.; Ni Mhurchu C.; Jiang Y.; Maddison R. | Modern screen-use behaviors: The effects of single- and multi-screen use on energy intake | 2015 |  |  |  |  | √ |
| 125 | Nooner K.; Schaefer L. | A pilot investigation of media multitasking and trauma symptoms among adolescents | 2015 |  |  |  |  | √ |
| 126 | Dias P. | Motivations for multi-screening: An exploratory study on motivations and gratifications | 2016 | √ |  |  |  |  |
| 127 | Yang X.; Zhu L. | Predictors of media multitasking in Chinese adolescents | 2016 | √ |  |  |  |  |
| 128 | Terry C. A.; Mishra P.; Roseth C. J. | Preference for multitasking, technological dependency, student metacognition, & pervasive technology use: An experimental intervention | 2016 |  |  | √ |  |  |
| 129 | Rubenking B. | Multitasking with TV: Media technology, genre, and audience influences | 2016 | √ |  |  |  |  |
| 130 | Srivastava J.; Nakazawa M.; Chen Y. W. | Online, mixed, and offline media multitasking: Role of cultural, socio-demographic, and media factors | 2016 | √ |  |  |  |  |
| 131 | Cain M. S.; Leonard J. A.; Gabrieli J. D. E.; Finn A. S. | Media multitasking in adolescence | 2016 |  | √ |  |  |  |
| 132 | Moisala M.; Salmela V.; Hietajärvi L.; Salo E.; Carlson S.; Salonen O.; Lonka K.; Hakkarainen K.; Salmela-Aro K.; Alho K. | Media multitasking is associated with distractibility and increased prefrontal activity in adolescents and young adults | 2016 |  | √ |  |  |  |
| 133 | Gorman T. E.; Green C. S. | Short-term mindfulness intervention reduces the negative attentional effects associated with heavy media multitasking | 2016 |  | √ |  |  |  |
| 134 | Uncapher M. R.; Thieu M. K.; Wagner A. D. | Media multitasking and memory: Differences in working memory and long-term memory | 2016 |  | √ |  |  |  |
| 135 | Cardoso-Leite P.; Kludt R.; Vignola G.; Ma W. J.; Green C. S.; Bavelier D. | Technology consumption and cognitive control: Contrasting action video game experience with media multitasking | 2016 |  | √ |  |  |  |
| 136 | Shin D. H., An H., Kim J. H. | How the second screens change the way people interact and learn: The effects of second screen use on information processing | 2016 |  |  | √ |  |  |
| 137 | Loh K. K.; Tan B. Z. H.; Lim S. W. H. | Media multitasking predicts video-recorded lecture learning performance through mind wandering tendencies | 2016 |  |  | √ |  |  |
| 138 | Calderwood C.; Green J. D.; Joy-Gaba J. A.; Moloney J. M. | Forecasting errors in student media multitasking during homework completion | 2016 |  |  | √ |  |  |
| 139 | Dindar, M.; Akbulut, Y. | Effects of multitasking on retention and topic interest | 2016 |  |  | √ |  |  |
| 140 | Gupta, N.; Irwin, J. D. | In-class distractions: The role of Facebook and the primary learning task | 2016 |  |  | √ |  |  |
| 141 | Kononova A.; Joo E.; Yuan S. | If I choose when to switch: Heavy multitaskers remember online content better than light multitaskers when they have the freedom to multitask | 2016 |  |  |  | √ |  |
| 142 | Jeong S. H.; Hwang Y. | Media multitasking effects on cognitive vs. attitudinal outcomes: A meta-analysis | 2016 |  |  |  | √ |  |
| 143 | Kazakova S.; Cauberghe V.; Hudders L.; Labyt C. | The impact of media multitasking on the cognitive and attitudinal responses to television commercials: The moderating role of type of advertising appeal | 2016 |  |  |  | √ |  |
| 144 | Segijn C. M.; Voorveld H. A. M.; Smit E. G. | The underlying mechanisms of multiscreening effects | 2016 |  |  |  | √ |  |
| 145 | Angell R.; Gorton M.; Sauer J.; Bottomley P.; White J. | Don’t distract me when I’m media multitasking: Toward a theory for raising advertising recall and recognition | 2016 |  |  |  | √ |  |
| 146 | Ran W.; Yamamoto M.; Xu S. | Media multitasking during political news consumption: A relationship with factual and subjective political knowledge | 2016 |  |  |  | √ |  |
| 147 | Kätsyri J.; Kinnunen T.; Kusumoto K.; Oittinen P.; Ravaja N. | Negativity bias in media multitasking: The effects of negative social media messages on attention to television news broadcasts | 2016 |  |  |  | √ |  |
| 148 | Xu S.; Wang Z.; David P. | Media multitasking and well-being of university students | 2016 |  |  |  |  | √ |
| 149 | Robinson H. R. | Multiple media use, polychronicity and multitasking: A review of literature and proposed research directions | 2016 | √ |  |  |  |  |
| 150 | Chang Y. | Why do young people multitask with multiple media? Explicating the relationships among sensation seeking, needs, and media multitasking behavior | 2017 | √ |  |  |  |  |
| 151 | Khan S. K.; Li G.; Raza A.; Ahmed J.; Khan I.; Ali N. | Factors influencing cross-screen engagement in young Chinese users | 2017 | √ |  |  |  |  |
| 152 | Robinson H. R. | Towards an enhanced understanding of the behavioural phenomenon of multiple media use | 2017 | √ |  |  |  |  |
| 153 | Kononova A. G.; Yuan S. | Take a break: Examining college students’ media multitasking activities and motivations during study-or work-related tasks | 2017 | √ |  |  |  |  |
| 154 | Baumgartner S. E.; Lemmens J. S.; Weeda W. D.; Huizinga M. | Measuring media multitasking: Development of a short measure of media multitasking for adolescents | 2017 | √ |  |  |  |  |
| 155 | Valecha P. | Multi-screening behavior of young Indians and its implications for programmers and advertisers | 2017 | √ |  |  | √ |  |
| 156 | Segijn C. M.; Voorveld H. A. M.; Vandeberg L.; Pennekamp S. F.; Smit E. G. | Insight into everyday media use with multiple screens | 2017 | √ |  |  |  |  |
| 157 | Brasel S. A.; Gips J. | Media multitasking: How visual cues affect switching behavior | 2017 |  | √ |  |  |  |
| 158 | Uncapher M. R.; Lin L.; Rosen L. D.; Kirkorian H. L.; Baron N. S.; Bailey K.; Cantor J.; Strayer D. L.; Parsons T. D.; Wagner A. D. | Media multitasking and cognitive, psychological, neural, and learning differences | 2017 |  | √ |  |  |  |
| 159 | Wiradhany W.; Nieuwenstein M. R. | Cognitive control in media multitaskers: Two replication studies and a meta-Analysis | 2017 |  | √ |  |  |  |
| 160 | Alzahabi R.; Becker M. W.; Hambrick D. Z. | Investigating the relationship between media multitasking and processes involved in task-switching | 2017 |  | √ |  |  |  |
| 161 | Murphy K.; McLauchlan S.; Lee M. | Is there a link between media-multitasking and the executive functions of filtering and response inhibition? | 2017 |  | √ |  |  |  |
| 162 | Baumgartner S. E.; Sumter S. R. | Dealing with media distractions: An observational study of computer-based multitasking among children and adults in the Netherlands | 2017 |  | √ |  |  |  |
| 163 | Edwards K. S.; Shin M. | Media multitasking and implicit learning | 2017 |  | √ |  |  |  |
| 164 | Steinborn M. B.; Huestegge L. | Phone conversation while processing information: Chronometric analysis of load effects in everyday-media multitasking | 2017 |  | √ |  |  |  |
| 165 | Ralph B. C. W.; Smilek D. | Individual differences in media multitasking and performance on the n-back | 2017 |  | √ |  |  |  |
| 166 | Magen H. | The relations between executive functions, media multitasking and polychronicity | 2017 |  | √ |  |  |  |
| 167 | Le Roux D. B.; Parry D. A. | In-lecture media use and academic performance: Does subject area matter? | 2017 |  |  | √ |  |  |
| 168 | Wu J. Y. | The indirect relationship of media multitasking self-efficacy on learning performance within the personal learning environment: Implications from the mechanism of perceived attention problems and self-regulation strategies | 2017 |  |  | √ |  |  |
| 169 | Lau W. W. F. | Effects of social media usage and social media multitasking on the academic performance of university students | 2017 |  |  | √ |  |  |
| 170 | Patterson M. C. | A naturalistic investigation of media multitasking while studying and the effects on exam performance | 2017 |  |  | √ |  |  |
| 171 | Segijn C. M.; Voorveld H. A. M.; Smit E. G. | How related multiscreening could positively affect advertising outcomes | 2017 |  |  |  | √ |  |
| 172 | Beuckels E.; Cauberghe V.; Hudders L. | How media multitasking reduces advertising irritation: The moderating role of the Facebook wall | 2017 |  |  |  | √ | √ |
| 173 | Rubenking B. | Boring is bad: Effects of emotional content and multitasking on enjoyment and memory | 2017 |  |  |  | √ |  |
| 174 | Kononova A.; Yuan S.; Joo E. | Reading about the flu online: How health-protective behavioral intentions are influenced by media multitasking, polychronicity, and strength of health-related arguments | 2017 |  |  |  | √ |  |
| 175 | Gottfried J. A.; Hardy B. W.; Holbert R. L.; Winneg K. M.; Jamieson K. H. | The changing nature of political debate consumption: Social media, multitasking, and knowledge acquisition | 2017 |  |  |  | √ |  |
| 176 | Nee R. C.; Dozier D. M. | Second screen effects: Linking multiscreen media use to television engagement and incidental learning | 2017 |  |  |  | √ |  |
| 177 | Segijn C. M.; Voorveld H. A. M.; Vandeberg L.; Smit E. G. | The battle of the screens: Unraveling attention allocation and memory effects when multiscreening | 2017 |  | √ |  | √ |  |
| 178 | Garaus M.; Wagner U.; Bäck A. M. | The effect of media multitasking on advertising message effectiveness | 2017 |  |  |  | √ |  |
| 179 | Segijn C. | Everyday multiscreening: How the simultaneous usage of multiple screens affects information processing and advertising effectiveness | 2017 |  |  |  | √ |  |
| 180 | Bellman S.; Robinson J. A.; Wooley B.; Varan D. | The effects of social TV on television advertising effectiveness | 2017 |  |  |  | √ |  |
| 181 | Chang Y. | The influence of media multitasking on the impulse to buy: A moderated mediation model | 2017 |  |  |  |  | √ |
| 182 | Robinson H. R. | Individuals’ preference for multiple media use–underlying motives | 2017 | √ |  |  |  |  |
| 183 | Robinson H. R.; Kalafatis S. P. | The ‘polychronicity-multiple media use’ (P-MMU) scale: A multi-dimensional scale to measure polychronicity in the context of multiple media use | 2017 | √ |  |  |  |  |
| 184 | Luo J.; Sun M.; Yeung P. S.; Li H. | Development and validation of a scale to measure media multitasking among adolescents: Results from China | 2018 | √ |  |  |  |  |
| 185 | Hwang Y.; Jeong S. H. | Multitasking and task performance: Roles of task hierarchy, sensory interference, and behavioral response | 2018 |  | √ |  |  |  |
| 186 | Segijn C. M.; Kononova A. | Audience, media, and cultural factors as predictors of multiscreen use: A comparative study of the Netherlands and the United States | 2018 | √ |  |  |  |  |
| 187 | Seddon A. L.; Law A. S.; Adams A. M.; Simmons F. R. | Exploring the relationship between executive functions and self-reported media-multitasking in young adults | 2018 |  | √ |  |  |  |
| 188 | Uncapher M. R.; Wagner A. D. | Minds and brains of media multitaskers: Current findings and future directions | 2018 |  | √ |  |  |  |
| 189 | Szumowska E.; Popławska-Boruc A.; Kuś J.; Osowiecka M.; Kramarczyk J. | When frequent media multitaskers perform worse and when they do not: The role of self-regulation ability and strategy manipulation | 2018 |  | √ |  |  |  |
| 190 | Baumgartner S. E.; Van Der Schuur W. A.; Lemmens J. S.; Te Poel F. | The relationship between media multitasking and attention problems in adolescents: Results of two longitudinal studies | 2018 |  | √ |  |  |  |
| 191 | May K. E.; Elder A. D. | Efficient, helpful, or distracting? A literature review of media multitasking in relation to academic performance | 2018 |  |  | √ |  |  |
| 192 | Guinness K. E.; Beaulieu L.; MacDonald J. M. | Effects of technology breaks on media multitasking with college students | 2018 |  |  | √ |  |  |
| 193 | Lin L.; Parsons T. D. | Ecologically valid assessments of attention and learning engagement in media multitaskers | 2018 |  |  | √ |  |  |
| 194 | Hayashi Y.; Blessington G. P. | A behavioral economic analysis of media multitasking: Delay discounting as an underlying process of texting in the classroom | 2018 |  |  | √ |  |  |
| 195 | Demirbilek M.; Talan T. | The effect of social media multitasking on classroom performance | 2018 |  |  | √ |  |  |
| 196 | Waite B. M.; Lindberg R.; Ernst B.; Bowman L. L.; Levine L. E. | Off-task multitasking, note-taking and lower- and higher-order classroom learning | 2018 |  |  | √ |  |  |
| 197 | Lee S.; Baek J.; Han G. | Effects of using a second-screen application on attention, learning, and user experience in an educational content | 2018 |  |  | √ |  |  |
| 198 | Lampi J. P.; Wilson N. E.; Armstrong S. L. | Complicating silence: A case study investigation of optimal student writing ecologies | 2018 |  |  | √ |  |  |
| 199 | Parry D. A.; Le Roux D. B. | In-lecture media use and academic performance: Investigating demographic and intentional moderators | 2018 |  |  | √ |  |  |
| 200 | Jensen J. A.; Walsh P.; Cobbs J. | The moderating effect of identification on return on investment from sponsor brand integration | 2018 |  |  |  | √ |  |
| 201 | Shukla S.; Sharma P. | Emotions and media multitasking behaviour among Indian college students | 2018 |  |  |  |  | √ |
| 202 | Lopez R. B.; Salinger J. M.; Heatherton T. F.; Wagner D. D. | Media multitasking is associated with altered processing of incidental, irrelevant cues during person perception | 2018 |  |  |  |  | √ |
| 203 | van der Schuur W. A.; Baumgartner S. E.; Sumter S. R.; Valkenburg P. M. | Media multitasking and sleep problems: A longitudinal study among adolescents | 2018 |  |  |  |  | √ |
| 204 | Hatchel T.; Negriff S.; Subrahmanyam K. | The relation between media multitasking, intensity of use, and well-being in a sample of ethnically diverse emerging adults | 2018 |  |  |  |  | √ |
| 205 | Hadlington L.; Murphy K. | Is media multitasking good for cybersecurity? Exploring the relationship between media multitasking and everyday cognitive failures on self-reported risky cybersecurity behaviors | 2018 |  |  |  |  | √ |
| 206 | Xu S.; David P. | Distortions in time perceptions during task switching | 2018 |  |  |  |  | √ |
| 207 | Kononova A.; McAlister A.; Oh H.J. | Screen overload: Pleasant multitasking with screen devices leads to the choice of healthful over less healthful snacks when compared with unpleasant multitasking | 2018 |  |  |  |  | √ |
| 208 | Limtrakul N.; Louthrenoo O.; Narkpongphun A.; Boonchooduang N.; Chonchaiya W. | Media use and psychosocial adjustment in children and adolescents | 2018 |  |  |  |  | √ |
| 209 | Oh C.; Herrera F.; Bailenson J. | The effects of immersion and real-world distractions on virtual social interactions | 2019 | √ |  |  |  | √ |
| 210 | Wiradhany W.; Koerts J. | Everyday functioning-related cognitive correlates of media multitasking: A mini meta-analysis | 2019 |  | √ |  |  | √ |
| 211 | Segijn C. M.; Xiong S.; Duff B. R. L. | Manipulating and measuring media multitasking: Implications of previous research and guidelines for future research | 2019 | √ |  |  |  |  |
| 212 | Aagaard J. | Multitasking as distraction: A conceptual analysis of media multitasking research | 2019 | √ |  |  |  |  |
| 213 | Wiradhany W.; Baumgartner S. E. | Exploring the variability of media multitasking choice behaviour using a network approach | 2019 | √ |  |  |  |  |
| 214 | Lin T. T. C. | Why do people watch multiscreen videos and use dual screening? Investigating users’ polychronicity, media multitasking motivation, and media repertoire | 2019 | √ |  |  |  |  |
| 215 | Xu S.; Wang Z.; Woods K. | Multitasking and dual motivational systems: A dynamic longitudinal study | 2019 | √ |  |  |  |  |
| 216 | Ji Q. | Exploring the motivations for live posting during entertainment television viewing | 2019 | √ |  |  |  |  |
| 217 | Su L.; Chen S. C. | Exploring the typology and impacts of audience gratifications gained from TV-smartphone multitasking | 2019 | √ |  |  |  |  |
| 218 | Ralph B. C. W.; Smith A. C.; Seli P.; Smilek D. | The relation between task-unrelated media multitasking and task-related motivation | 2019 | √ |  |  |  |  |
| 219 | Hwang Y.; Jeong S. H. | The role of user control in media multitasking effects | 2019 |  | √ |  |  |  |
| 220 | Ralph B. C. W.; Smith A. C.; Seli P.; Smilek D. | Yearning for distraction: Evidence for a trade-off between media multitasking and mind wandering | 2019 | √ |  |  |  |  |
| 221 | Hayashi Y.; Nenstiel J. N. | Media multitasking in the classroom: Problematic mobile phone use and impulse control as predictors of texting in the classroom | 2019 | √ |  | √ |  |  |
| 222 | Guo M. | Social television viewing with second screen platforms: Antecedents and consequences | 2019 | √ |  |  | √ |  |
| 223 | Shin M.; Webb A.; Kemps E. | Media multitasking, impulsivity and dual task ability | 2019 |  | √ |  |  |  |
| 224 | Parry D. A.; Le Roux D. B. | Media multitasking and cognitive control: A systematic review of interventions | 2019 |  | √ |  |  |  |
| 225 | Elbe P.; Sörman D. E.; Mellqvist E.; Brändström J.; Ljungberg J. K. | Predicting attention shifting abilities from self-reported media multitasking | 2019 |  | √ |  |  |  |
| 226 | Brown A.; Aizpurua A.; Jay C.; Evans M.; Glancy M.; Harper S. | Contrasting delivery modes for second screen TV content—push or pull? | 2019 |  | √ |  |  |  |
| 227 | Le Roux D. B.; Parry D. A. | Investigating differences in the attention distribution strategies of high and low media multitaskers through a two-dimensional game | 2019 |  | √ |  |  |  |
| 228 | Law A. S.; Stock R. | Learning approach and its relationship to type of media use and frequency of media-multitasking | 2019 |  |  | √ |  |  |
| 229 | Wammes J. D.; Ralph B. C. W.; Mills C.; Bosch N.; Duncan T. L.; Smilek D. | Disengagement during lectures: Media multitasking and mind wandering in university classrooms | 2019 |  |  | √ |  |  |
| 230 | Martín-Perpiñá M. M.; Poch F. V.; Cerrato S. M. | Media multitasking impact in homework, executive function and academic performance in Spanish adolescents | 2019 |  |  | √ |  |  |
| 231 | Liu Y.; Gu X. | Media multitasking, attention, and comprehension: A deep investigation into fragmented reading | 2019 |  |  | √ |  |  |
| 232 | Wu J. Y.; Cheng T. | Who is better adapted in learning online within the personal learning environment? Relating gender differences in cognitive attention networks to digital distraction | 2019 |  |  | √ |  |  |
| 233 | Segijn C. M.; Eisend M. | A meta-analysis into multiscreening and advertising effectiveness: Direct effects, moderators, and underlying mechanisms | 2019 |  |  |  | √ |  |
| 234 | Beuckels E.; Kazakova S.; Cauberghe V.; Hudders L.; De Pelsmacker P. | Freedom makes you lose control: Executive control deficits for heavy versus light media multitaskers and the implications for advertising effectiveness | 2019 |  | √ |  | √ |  |
| 235 | Ran W.; Yamamoto M. | Media multitasking, second screening, and political knowledge: Task-relevant and task-irrelevant second screening during election news consumption | 2019 |  |  |  | √ |  |
| 236 | Guitart I. A.; Hervet G.; Hildebrand D. | Using eye-tracking to understand the impact of multitasking on memory for banner ads: The role of attention to the ad | 2019 |  |  |  | √ |  |
| 237 | Duff B. R. L.; Segijn C. M. | Advertising in a media multitasking era: Considerations and future directions | 2019 |  |  |  | √ |  |
| 238 | Liu G.; Fang L.; Pan Y.; Zhang D. | Media multitasking and adolescents’ sleep quality: The role of emotional-behavioral problems and psychological suzhi | 2019 |  |  |  |  | √ |
| 239 | Park S.; Xu X.; Rourke B.; Bellur S. | Do you enjoy TV, while tweeting? Effects of multitasking on viewers’ transportation, emotions and enjoyment | 2019 |  |  |  |  | √ |
| 240 | Lopez R. B.; Heatherton T. F.; Wagner D. D. | Media multitasking is associated with higher risk for obesity and increased responsiveness to rewarding food stimuli | 2019 |  |  |  |  | √ |
| 241 | Lopez R. B.; Brand J.; Gilbert-Diamond D. | Media multitasking is associated with higher body mass index in pre-adolescent children | 2019 |  |  |  |  | √ |
| 242 | Lee M.; Murphy K.; Andrews G. | Using media while interacting face-to-face is associated with psychosocial well-being and personality traits | 2019 |  |  |  |  | √ |
| 243 | Luo J.; Yeung P. S.; Li H. | The relationship among media multitasking, academic performance and self-esteem in Chinese adolescents: The cross-lagged panel and mediation analyses | 2020 |  |  | √ |  | √ |
| 244 | Srisinghasongkram P.; Trairatvorakul P.; Maes M.; Chonchaiya W. | Effect of early screen media multitasking on behavioural problems in school-age children | 2020 |  | √ |  |  | √ |
| 245 | Tamir I. | Whatsappsport: Using whatsapp while viewing sports events | 2020 | √ |  |  |  | √ |
| 246 | Wiradhany W.; van Vugt M. K.; Nieuwenstein M. R. | Media multitasking, mind-wandering, and distractibility: A large-scale study | 2020 |  | √ |  |  |  |
| 247 | Sun T.; Zhong B. | Multitasking as multisensory behavior: Revisiting media multitasking in the perspective of media ecology theory | 2020 | √ |  |  |  |  |
| 248 | Khan S. K.; Guoxin L. | Adoption of multi-screen multitasking in young generation of China: A perspective of self-regulation | 2020 | √ |  |  |  |  |
| 249 | Parry D. A.; Le Roux D. B.; Bantjes J. R. | Testing the feasibility of a media multitasking self-regulation intervention for students: Behaviour change, attention, and self-perception | 2020 |  | √ |  |  |  |
| 250 | van der Schuur W. A.; Baumgartner S. E.; Sumter S. R.; Valkenburg P. M. | Exploring the long-term relationship between academic-media multitasking and adolescents’ academic achievement | 2020 |  |  | √ |  |  |
| 251 | Liu Y.; Zhou S.; Zhang H. | Second screening use and its effect on political involvement in China: An integrated communication mediation model | 2020 |  |  |  | √ |  |
| 252 | Murphy K.; Creux O. | Examining the association between media multitasking, and performance on working memory and inhibition tasks | 2020 |  | √ |  |  |  |
| 253 | Höhne J. K.; Schlosser S.; Couper M. P.; Blom A. G. | Switching away: Exploring on-device media multitasking in web surveys | 2020 | √ |  |  |  |  |
| 254 | Shin M.; Linke A.; Kemps E. | Moderate amounts of media multitasking are associated with optimal task performance and minimal mind wandering | 2020 |  | √ |  |  |  |
| 255 | Mrazek A. J.; Mrazek M. D.; Carr P. C.; Delegard A. M.; Ding M. G.; Garcia D. I.; Greenstein J. E.; Kirk A. C.; Kodama E. E.; Krauss M. J.; Landry A. P.; Stokes C. A.; Wickens K. D.; Wong K.; Schooler J. W. | The feasibility of attention training for reducing mind-wandering and digital multitasking in high schools | 2020 |  | √ | √ |  |  |
| 256 | Hall A. C. G.; Lineweaver T. T.; Hogan E. E.; O’Brien S. W. | On or off task: The negative influence of laptops on neighboring students’ learning depends on how they are used | 2020 |  |  | √ |  |  |
| 257 | Raza M. Y.; Khan A. N.; Khan N. A.; Ali A.; Bano S. | Dark side of social media and academic performance of public sector schools students: Role of parental school support | 2020 |  |  | √ |  |  |
| 258 | Luo J.; Yeung P. S.; Li H. | Relationship between media multitasking and self-esteem among Chinese adolescents: Mediating roles of peer influence and family functioning | 2020 |  |  |  |  | √ |
| 259 | Lopez R. B.; Heatherton T. F.; Wagner D. D. | Media multitasking is associated with higher risk for obesity and increased responsiveness to rewarding food stimuli | 2020 |  |  |  |  | √ |
| 260 | Su L.; Chen S. C. | Exploring the typology and impacts of audience gratifications gained from TV–smartphone multitasking | 2020 | √ |  |  |  |  |
| 261 | Różańska A.; Gruszka A. | Current research trends in multitasking: A bibliometric mapping approach | 2020 |  | √ | √ |  |  |
| 262 | Billings A. C.; Lewis M.; Brown K. A.; Xu Q. | Top rated on five networks-and nearly as many devices: The NFL, social TV, fantasy sport, and the ever-present second screen | 2020 | √ |  |  |  |  |
| 263 | Ralph B. C. W.; Seli P.; Wilson K. E.; Smilek D. | Volitional media multitasking: Awareness of performance costs and modulation of media multitasking as a function of task demand | 2020 |  | √ |  |  |  |
| 264 | Liu Y.; Gu X. | Media multitasking, attention, and comprehension: A deep investigation into fragmented reading | 2020 |  |  | √ |  |  |
| 265 | Rogobete D. A.; Ionescu T.; Miclea M. | The relationship between media multitasking behavior and executive function in adolescence: A replication study | 2020 |  | √ |  |  |  |
| 266 | Shin M.; Kemps E. | Media multitasking as an avoidance coping strategy against emotionally negative stimuli | 2020 |  |  |  |  | √ |
| 267 | Rodriguez-Triana M. J.; Prieto L.; Holzer A.; Gillet D. | Instruction, student engagement and learning outcomes: A case study using anonymous social media in a face-to-face classroom | 2020 |  |  | √ |  |  |
| 268 | Yang C. C.; Christofferson K. | On the phone when we’re hanging out: Digital social multitasking (DSMT) and its socioemotional implications | 2020 |  |  |  |  | √ |
| 269 | Hartley K.; Bendixen L. D.; Olafson L.; Gianoutsos D.; Shreve E. | Development of the smartphone and learning inventory: Measuring self-regulated use | 2020 | √ |  |  |  |  |
| 270 | Ettinger K.; Cohen A. | Patterns of multitasking behaviours of adolescents in digital environments | 2020 | √ |  |  |  |  |
| 271 | Khan S. K.; Ali N.; Khan N. A.; Ammara U.; Anjum N. | Understanding multiscreening phenomenon for online shopping through perspective of self-regulation and dual process theory: Case of Chinese young generation | 2020 | √ |  |  |  |  |
| 272 | Kostyrka-Allchorne K.; Cooper N. R.; Simpson A.; Sonuga-Barke E. J. S. | Children’s mental health and recreation: Limited evidence for associations with screen use | 2020 |  |  |  |  | √ |
| 273 | Hoeck L.; Spann M. | An experimental analysis of the effectiveness of multi-screen advertising | 2020 |  |  |  | √ |  |
| 274 | Guo M. | Second screening: Measuring second screen user behavior in a social television viewing environment | 2020 | √ |  |  |  |  |
| 275 | Furini M.; De Michele R. | On improving the engagement between viewers and TV commercials through gamification | 2020 |  |  |  | √ |  |
| 276 | Segijn C. M.; Araujo T.; Voorveld H. A. M.; Smit E. G. | Related multiscreening as a strategy to retain audiences and increase persuasion during a commercial break | 2020 |  |  |  | √ |  |
| 277 | Sezen E.; Tsekleves E.; Mauthe A. | Bar charts versus plain numbers: Visualizations for enhancing the soccer-watching experience on TV via a second screen | 2020 |  |  |  | √ |  |
| 278 | Weimann-Saks D.; Ariel Y.; Elishar-Malka V. | Social second screen: Whatsapp and watching the world cup | 2020 |  |  |  | √ |  |
| 279 | Unkel J.; Kümpel A. S. | (A)synchronous communication about TV series on social media: A multi-method investigation of reddit discussions | 2020 |  |  |  | √ |  |
| 280 | Brown-Devlin N.; Devlin M. B.; Billings A. C.; Brown K. A. | Five rings, five screens? A global examination of social TV influence on social presence and social identification during the 2018 winter Olympic games | 2020 |  |  |  | √ |  |
| 281 | Robinson H. R.; Kalafatis S. P. | Why do people choose to multitask with media? The dimensions of polychronicity as drivers of multiple media use—A user typology | 2020 | √ |  |  |  |  |

Topic 1: Motivating and predicting media multitasking behaviors; Topic 2: Media multitasking and cognitive outcomes; Topic 3: Media multitasking and learning and academic performance; Topic 4: Media multitasking and information consumption; Topic 5: Media multitasking and socioemotional functions.
